# Supplementary material for: Promoting water dissociation for efficient solar driven CO2 electroreduction via improving hydroxyl adsorption
Source: Nat Commun. 2023 Feb 10;14:751. doi: 10.1038/s41467-023-36263-z (PMC9918482; doi:10.1038/s41467-023-36263-z)
Supplement: Supplementary file 1 — Supplementary Information [file 41467_2023_36263_MOESM1_ESM.pdf]

Supplementary Information

Promoting water dissociation for efficient solar driven CO_2 electroreduction via improving hydroxyl adsorption

Author Information

Xin Chen[1], Junxiang Chen[2], Huayu Chen[3], Qiqi Zhang[1], Jiaxuan Li[1], Jiwei Cui[1], Yanhui Sun[1], Defa Wang[1], Jinhua Ye[1,4] & Lequan Liu[1]*

[1] TJU-NIMS International Collaboration Laboratory, School of Materials Science and Engineering, Key Lab of Advanced Ceramics and Machining Technology (Ministry of Education), Tianjin University, Tianjin, P. R. China.

[2] CAS Key Laboratory of Design and Assembly of Functional Nanostructures, Fujian Key Laboratory of Nanomaterials, Fujian Institute of Research on the Structure of Matter, Chinese Academy of Sciences, Fuzhou, P. R. China.

[3] College of Materials and Chemistry, China Jiliang University, Hangzhou, P. R. China.

[4] International Center for Materials Nanoarchitectonics (WPI-MANA), National Institute for Materials Science (NIMS), 1-1 Namiki, Tsukuba, Japan.

*Corresponding author

Correspondence to: Lequan Liu

email: Lequan.Liu@tju.edu.cn

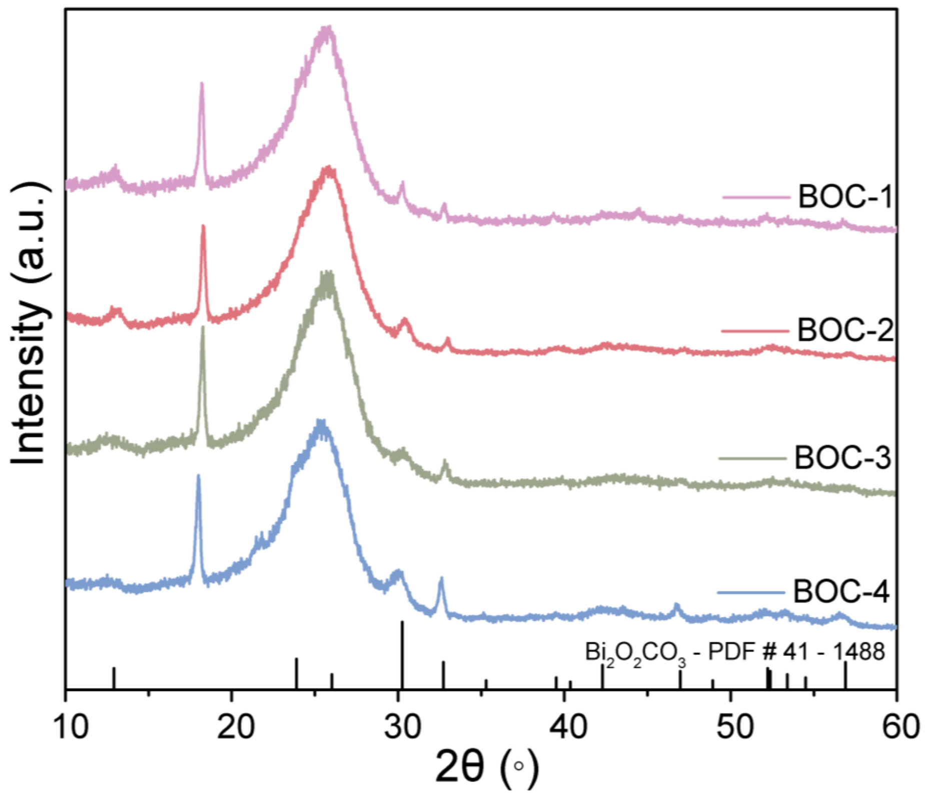

Supplementary Fig. 1 XRD patterns of different BOC samples.

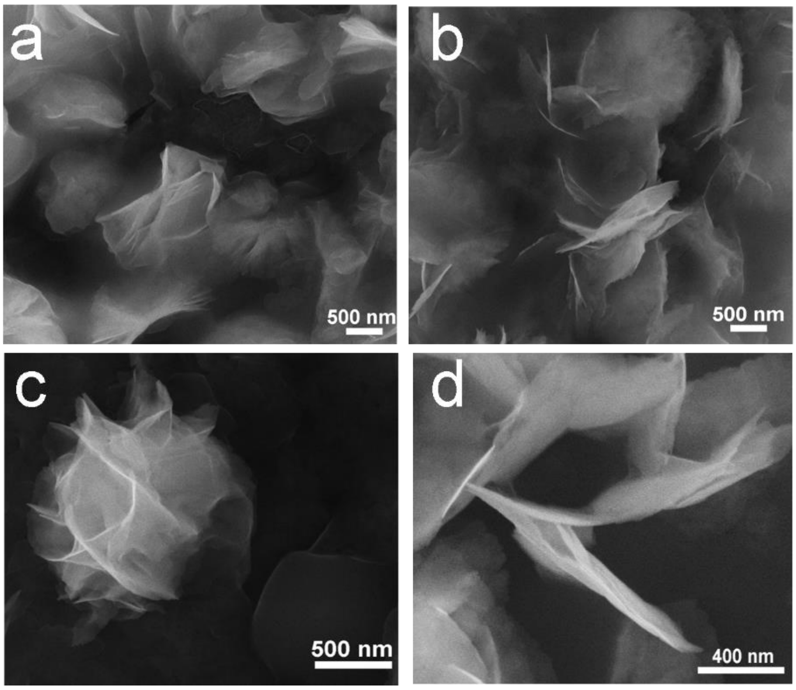

Supplementary Fig. 2 SEM characterization of different samples. SEM images of a) BOC-1, b) BOC-2, c) BOC-3 and d) BOC-4. All the BOC samples were grown on carbon as vertical nanosheets.

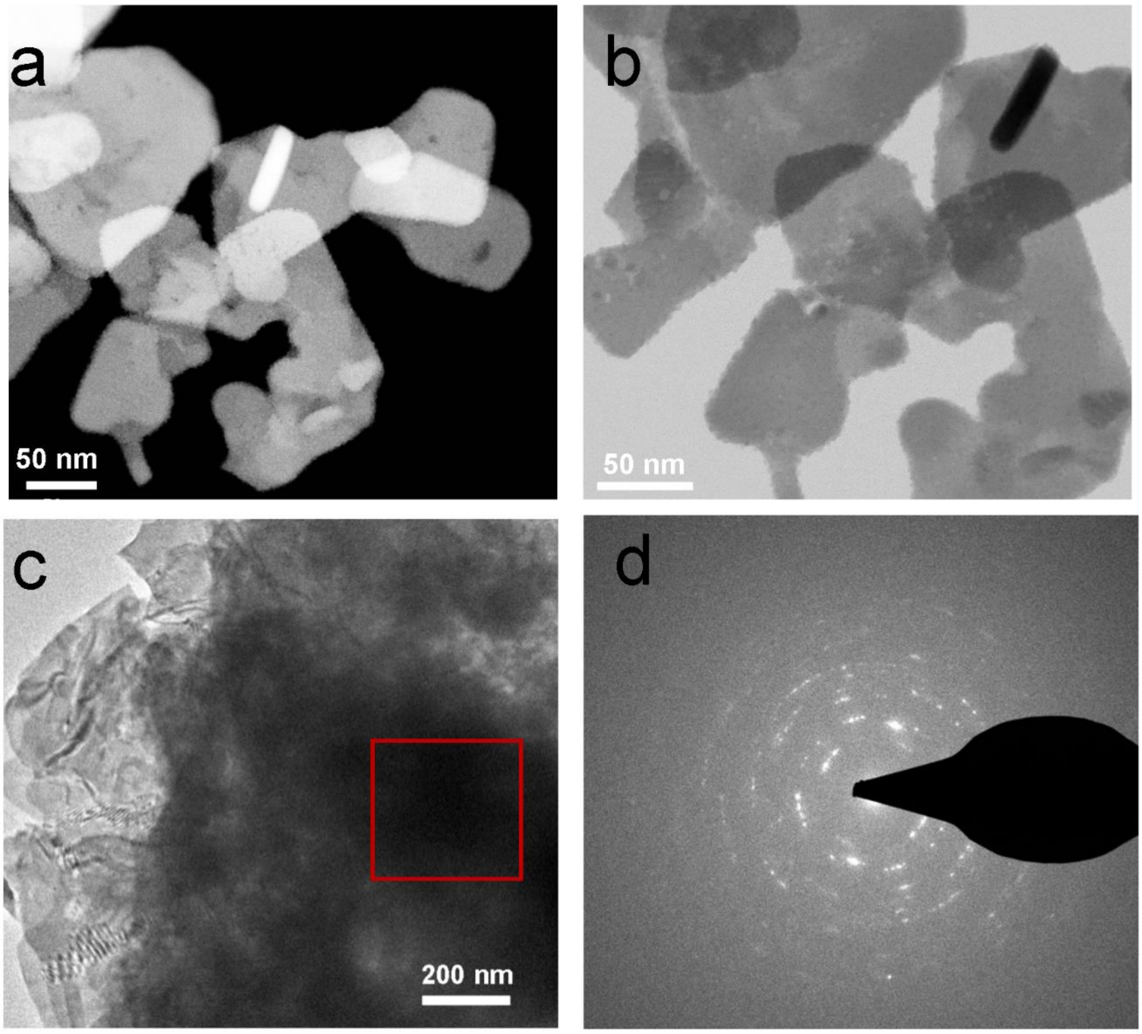

Supplementary Fig. 3 TEM characterization of BOC-2. a), b) and c)TEM images of BOC-2. d) The corresponding selected area electron diffraction for BOC-2 as circled in c.

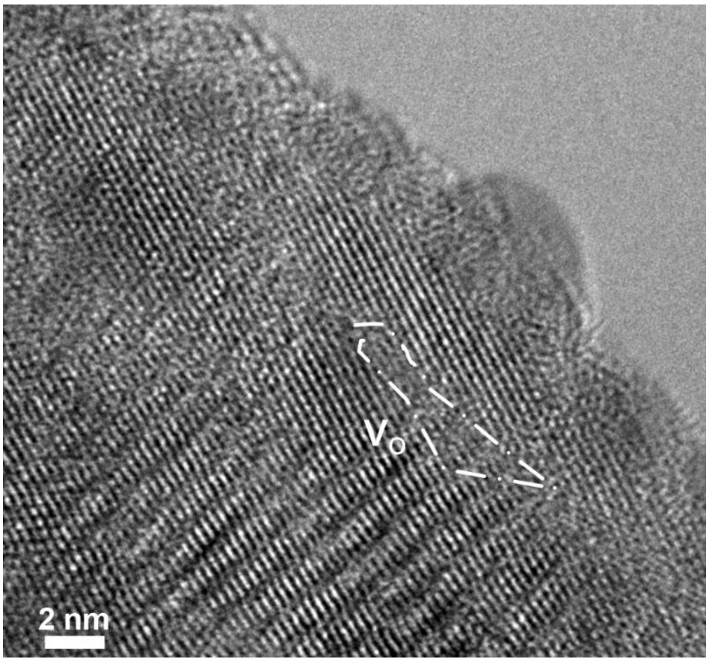

Supplementary Fig. 4 HRTEM image of BOC-2. The area circled by dashed line shows the distortion in lattice fringes.

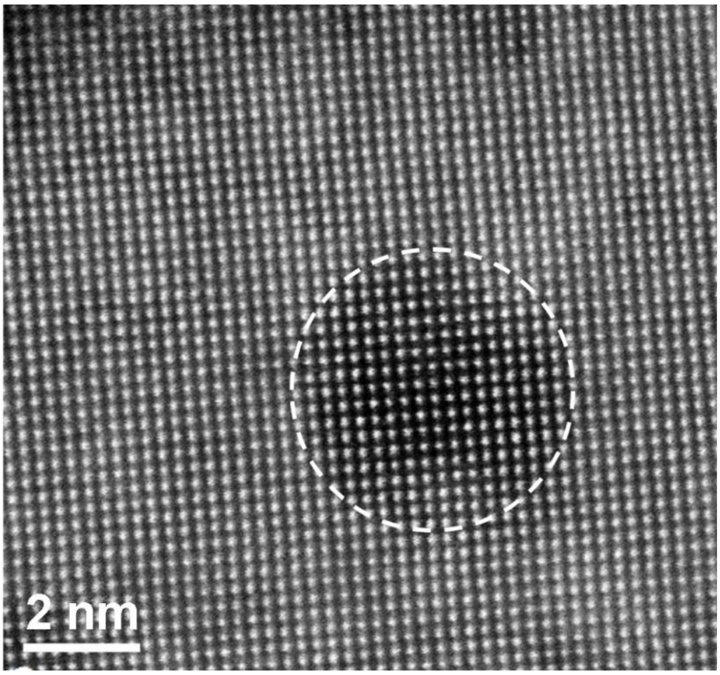

Supplementary Fig. 5 HADDF-STEM image of BOC-2. The area circled in dashed line shows that there are some local distortions in lattice by observing the atoms with different intensity.

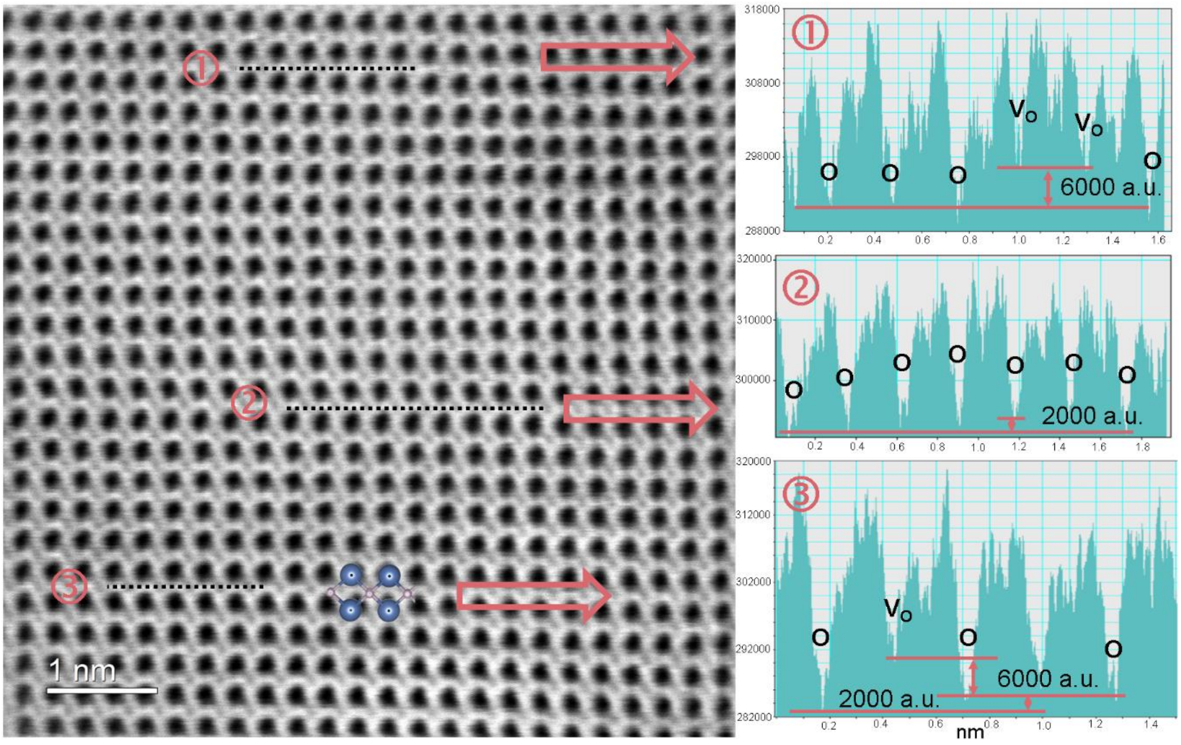

Supplementary Fig. 6 ABF-STEM image and the corresponding intensity profile of BOC-2. The blue balls represent bismuth while the purple balls represent oxygen. To eliminate the effect of error, three sections were selected randomly. Section 1 and 3 contain the oxygen vacancy and oxygen sites while there are only oxygen sites in section 2. The intensity of oxygen atom is similar, with a maximum difference of 2000 a.u. (section 2). In contrast, the difference between oxygen vacancy and oxygen atom is more than 6000 a.u., much larger than the error of oxygen atom intensity (section 1 and section 3). Therefore, the identification of V_O from STEM is relatively reliable.

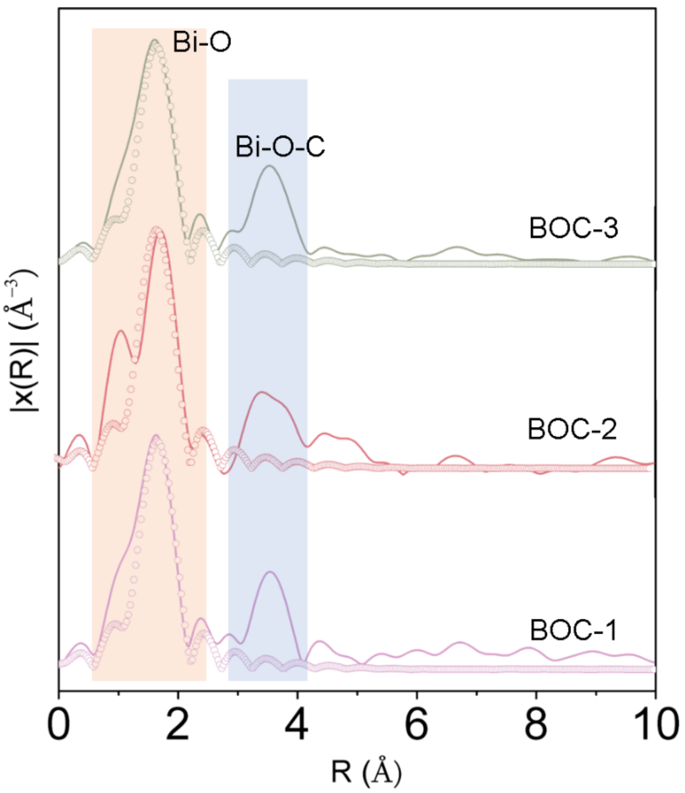

Supplementary Fig. 7 Fourier transform of Bi L_3 edge EXAFS data recorded at R space for BOC-1, BOC-2 and BOC-3.

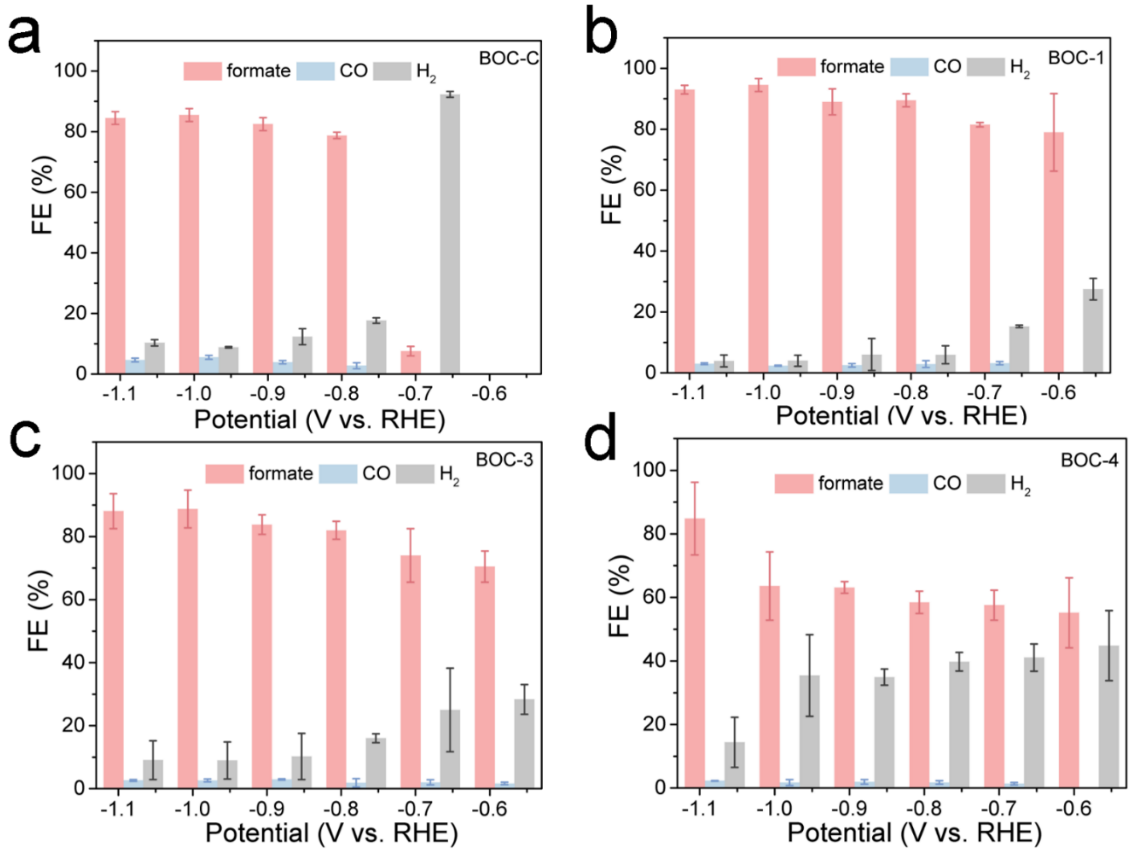

Supplementary Fig. 8 The Faradaic efficiency of different products as the function of potential for a) BOC-C, b) BOC-1, c) BOC-3 and d) BOC-4. The error bars represent the standard deviation of three independent experiments.

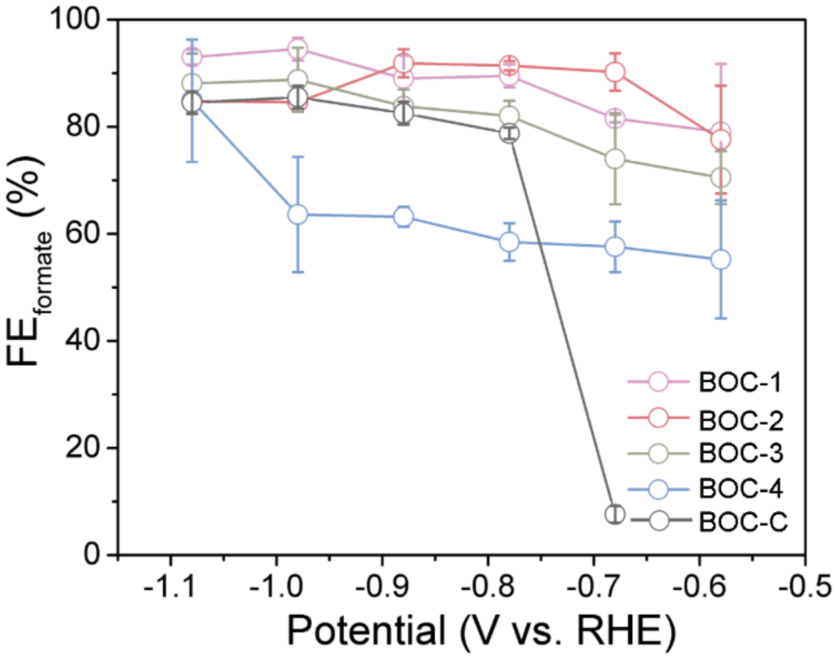

Supplementary Fig. 9 The Faradaic efficiency of formate at different applied potential for CO_2RR on BOC-C, BOC-1, BOC-2, BOC-3 and BOC-4. The error bars represent the standard deviation of three independent experiments.

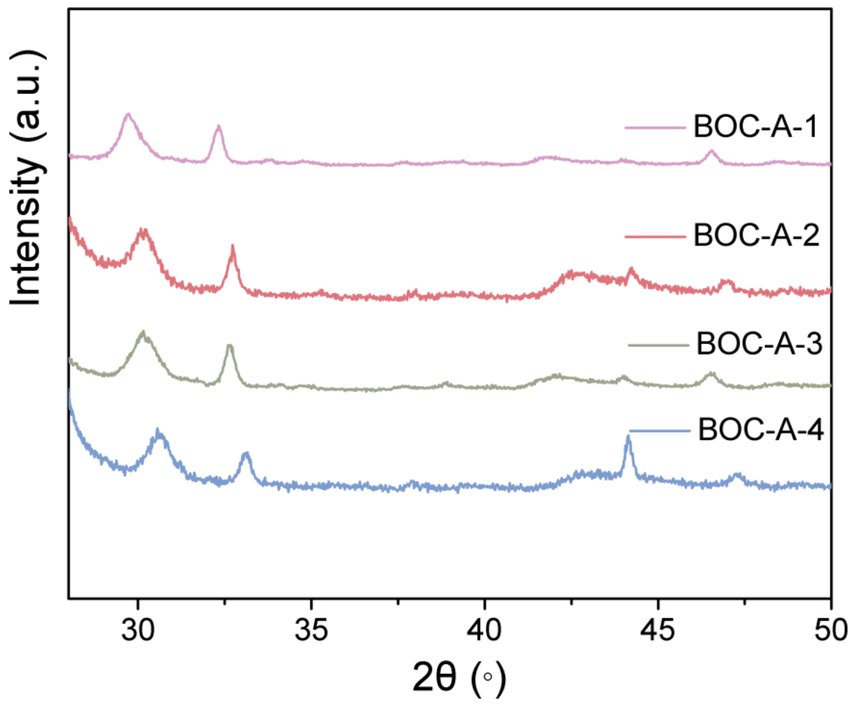

Supplementary Fig. 10 XRD patterens of BOC with annealing (BOC-A).

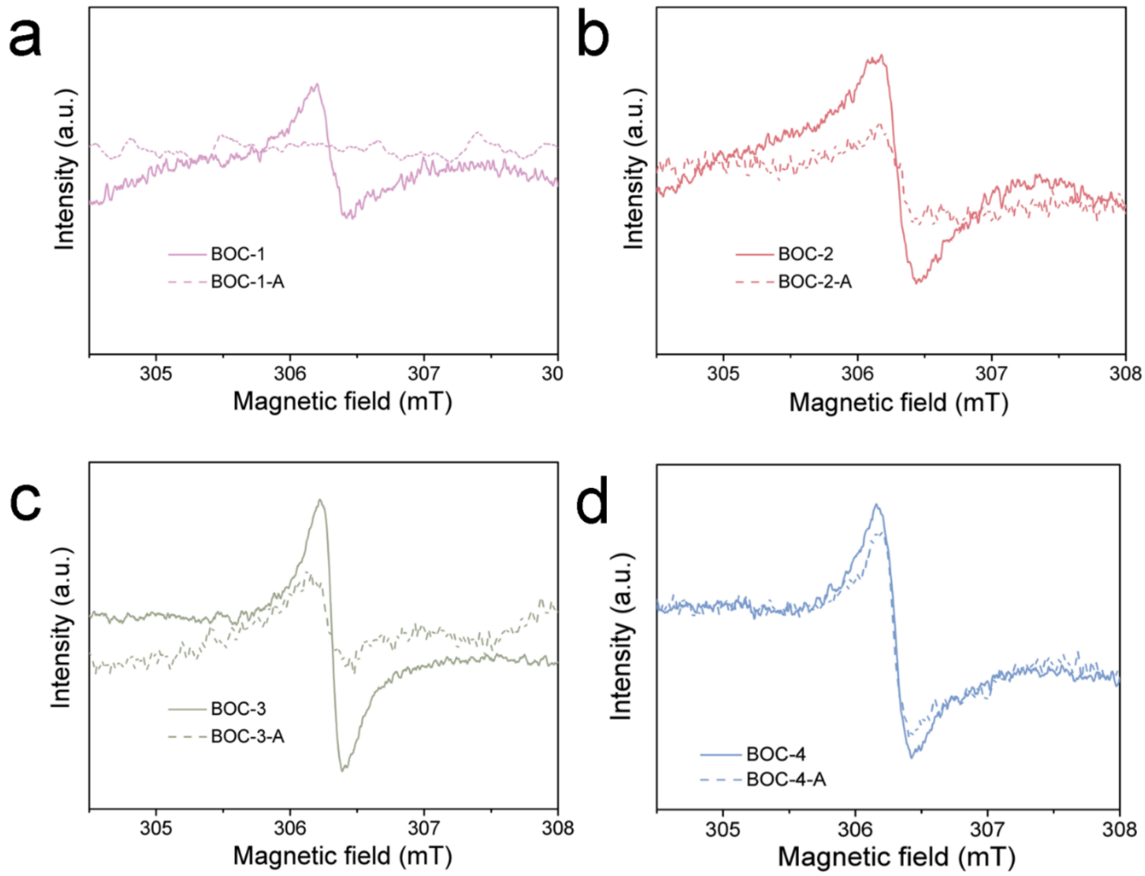

Supplementary Fig. 11 The EPR spectra of a) BOC-1, b) BOC-2, c) BOC-3 and d) BOC-4 before (solid line) and after (dashed line) annealing. It's obvious that the concentration of V_O in all BOC samples decreased after thermal treament, demonstrating that V_O are partially filled through annealing.

.

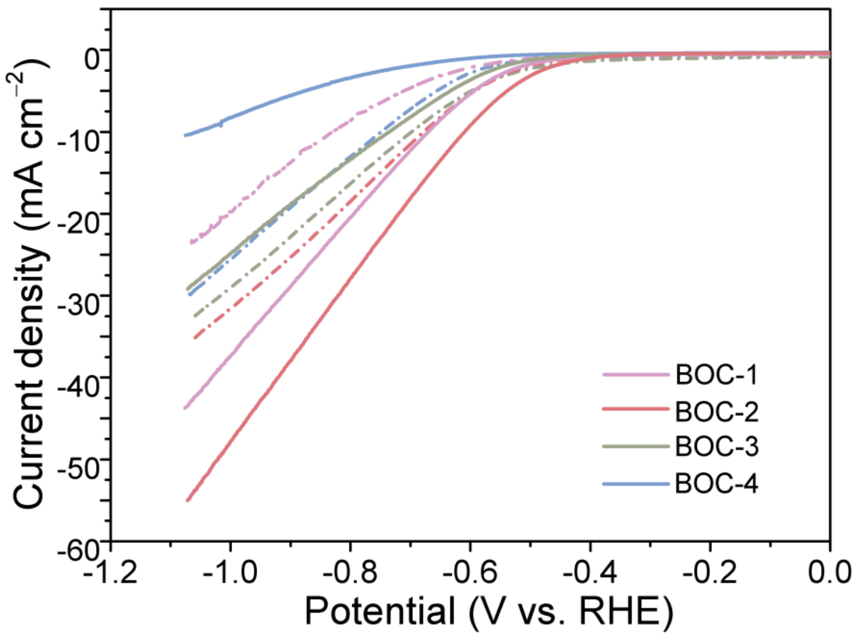

Supplementary Fig. 12 LSV curves of BOC-1, BOC-2, BOC-3, and BOC-4 before (solid line) and after (dashed line) annealing with the electrolyte of 0.5 M $KHCO_3$.

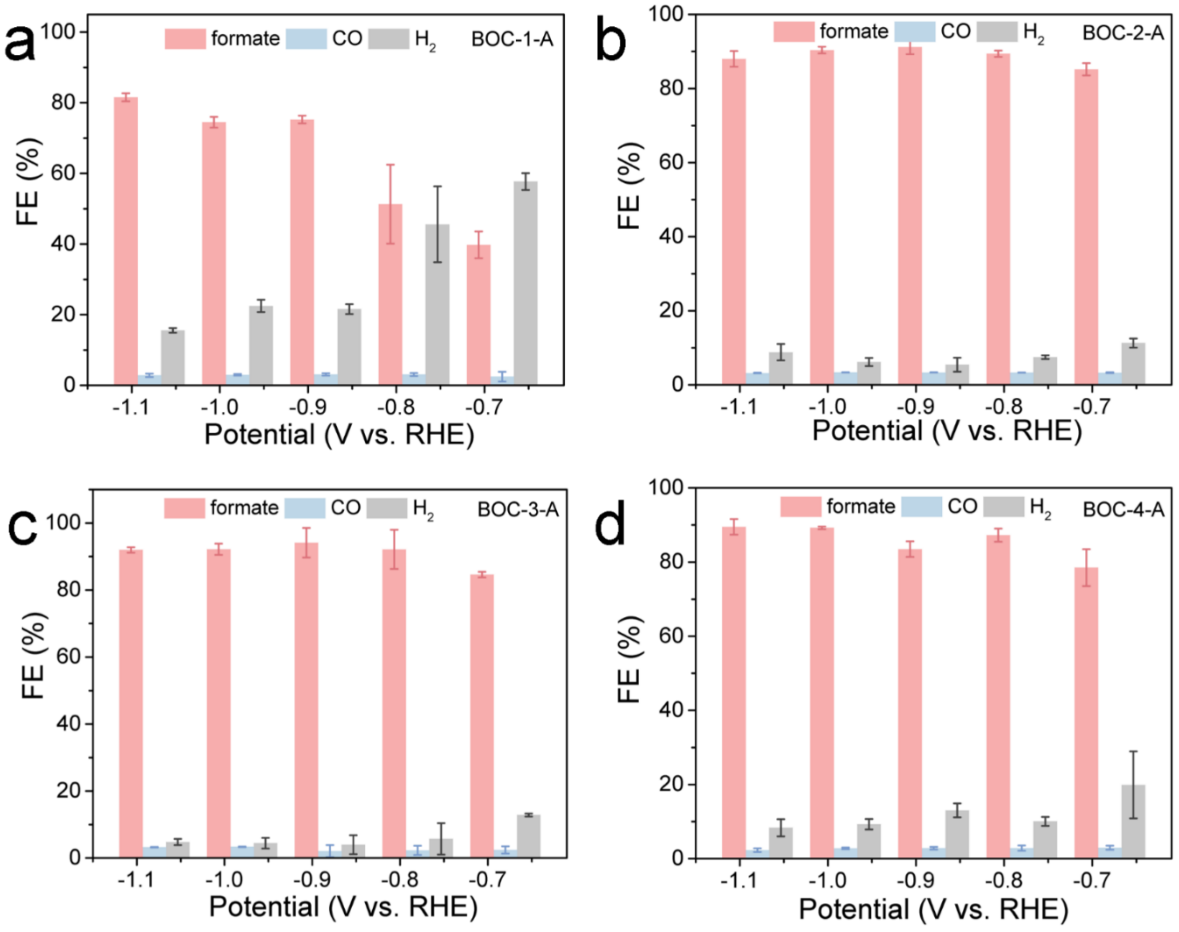

Supplementary Fig. 13 FE of formate, CO and H_2 as the function of potential for a) BOC-1-A, b) BOC-2-A, c) BOC-3-A and d) BOC-4-A. The error bars represent the standard deviation of three independent experiments.

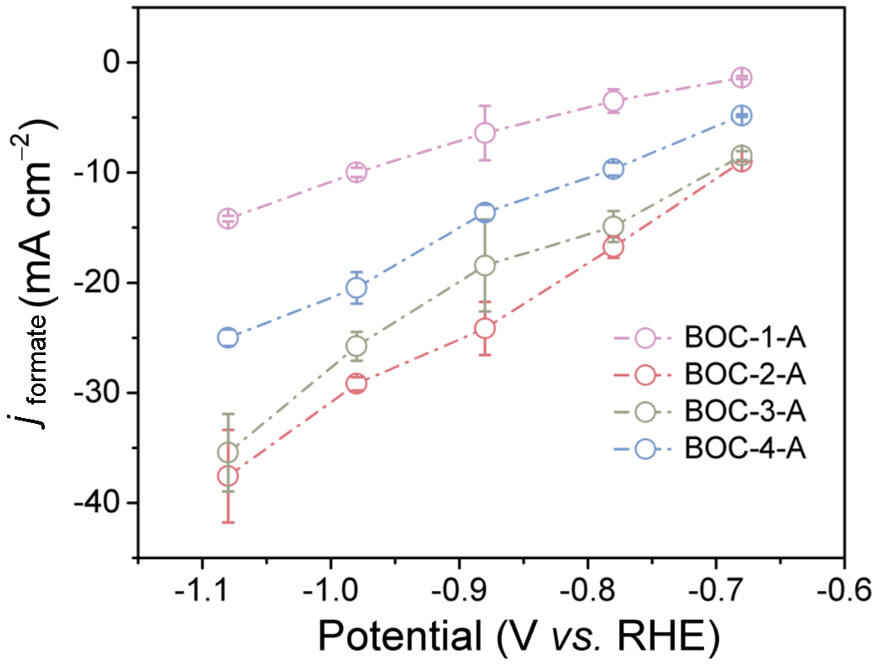

Supplementary Fig. 14 $j_{formate}$ of BOC after thermal treatment as function of applied potentials.

The error bars represent the standard deviation of three independent experiments.

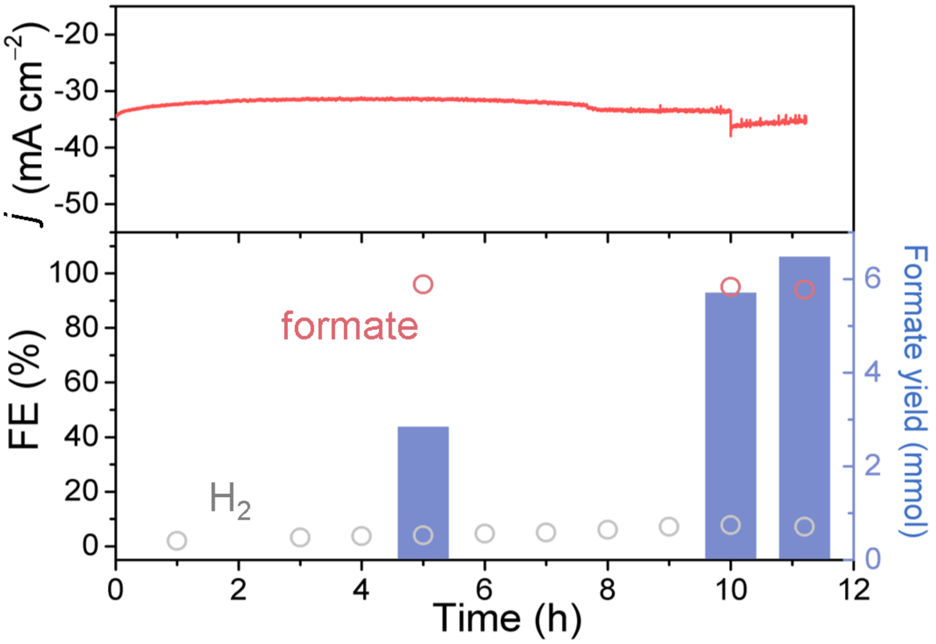

Supplementary Fig. 15 Stability test of BOC-2 at −0.88 V in H-cell.

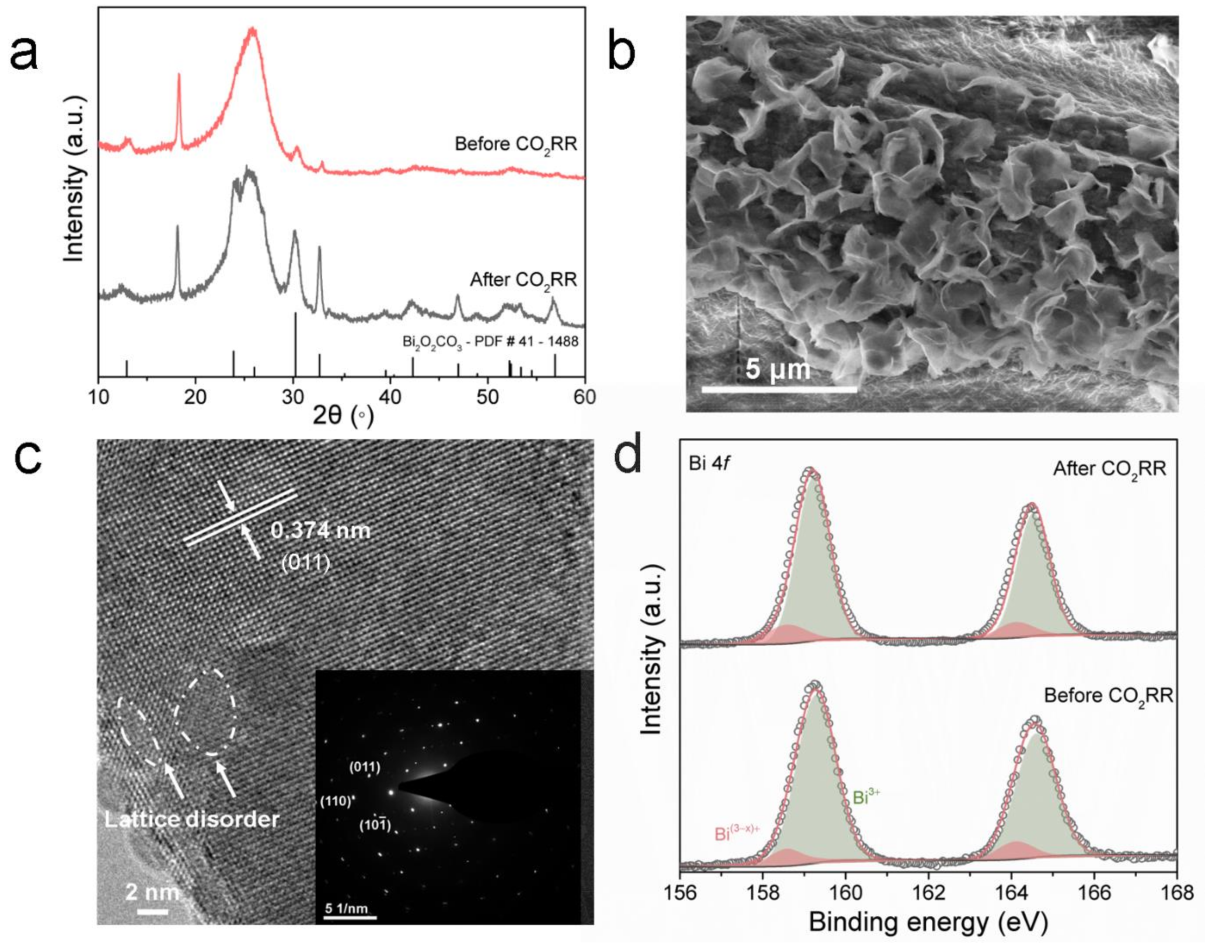

Supplementary Fig. 16 Phase and chemical state characterizations of BOC-2 after stability test.

a) XRD patterns, b) SEM image c) TEM and correponsponding selected area electron diffraction images and d) XPS spectra of Bi 4*f* for BOC after stability test.

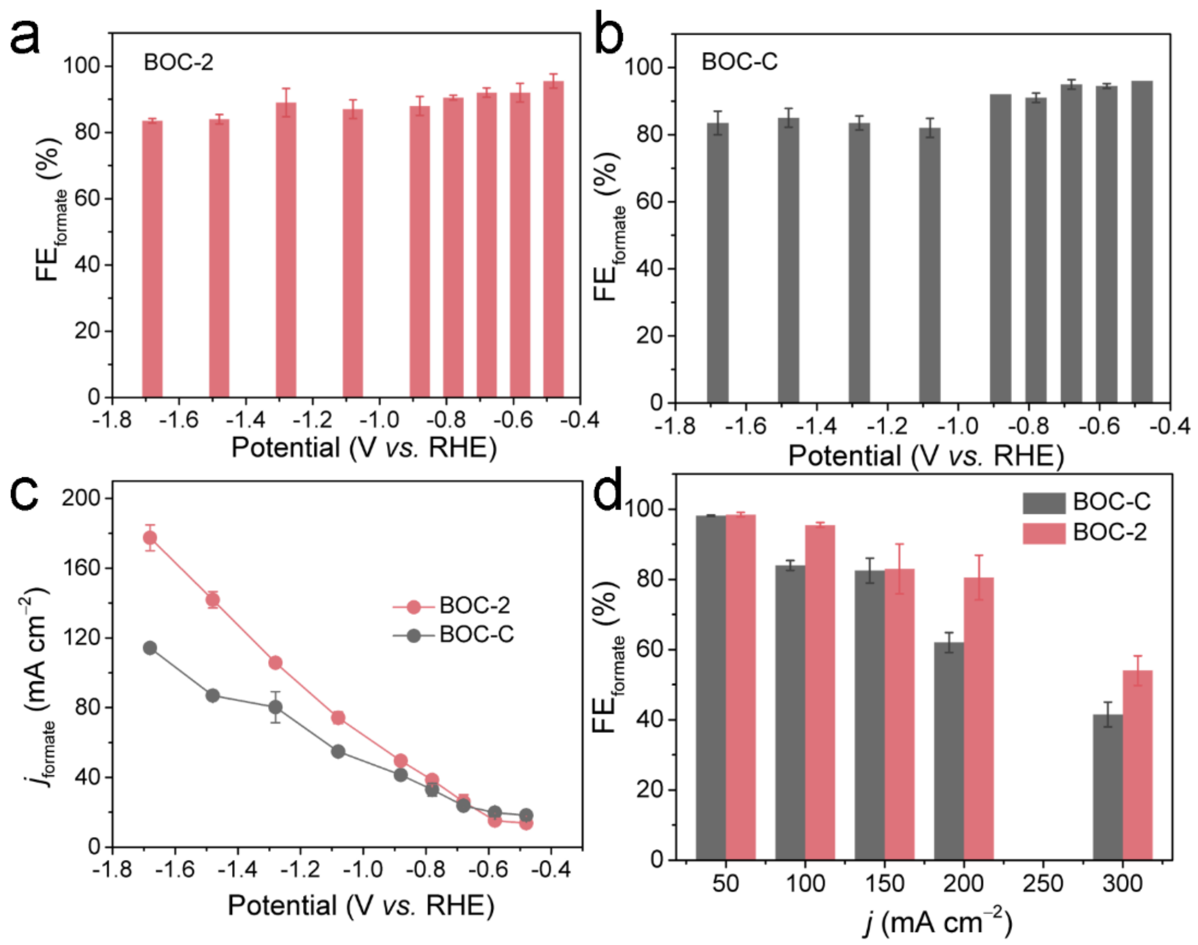

Supplementary Fig. 17 The performance of BOC-2 and BOC-C in flow-cell system. The $FE_{formate}$ of a) BOC-2 and b) BOC-C in flow-cell systems at different negative potentials. c) the partial current density of BOC-2 and BOC-C in flow-cell. d) The $FE_{formate}$ of BOC-2 and BOC-C at different current density. The error bars represent the standard deviation of three independent measurements.

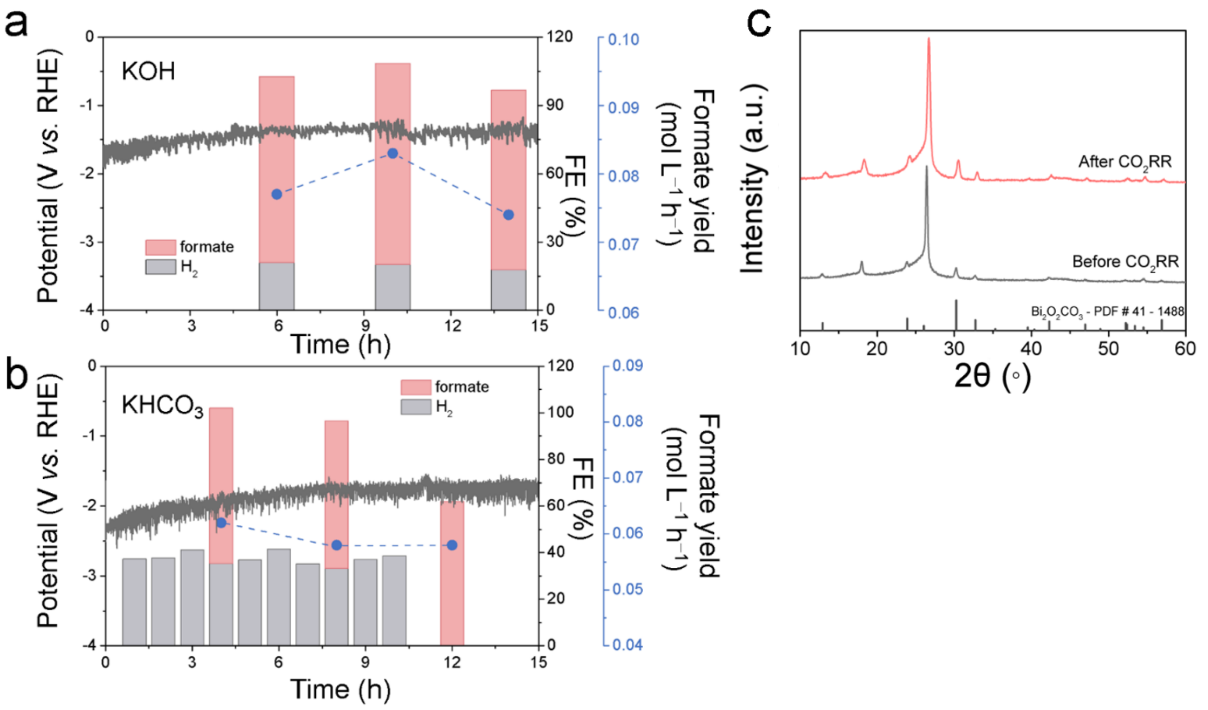

Supplementary Fig. 18 The stability test of BOC-2 at the current density of 200 mA cm^{-2} with a) 1 M KOH and b) 1 M $KHCO_3$ as electrolyte. c) The XRD patterns of BOC-2 before and after stability test in flow cell.

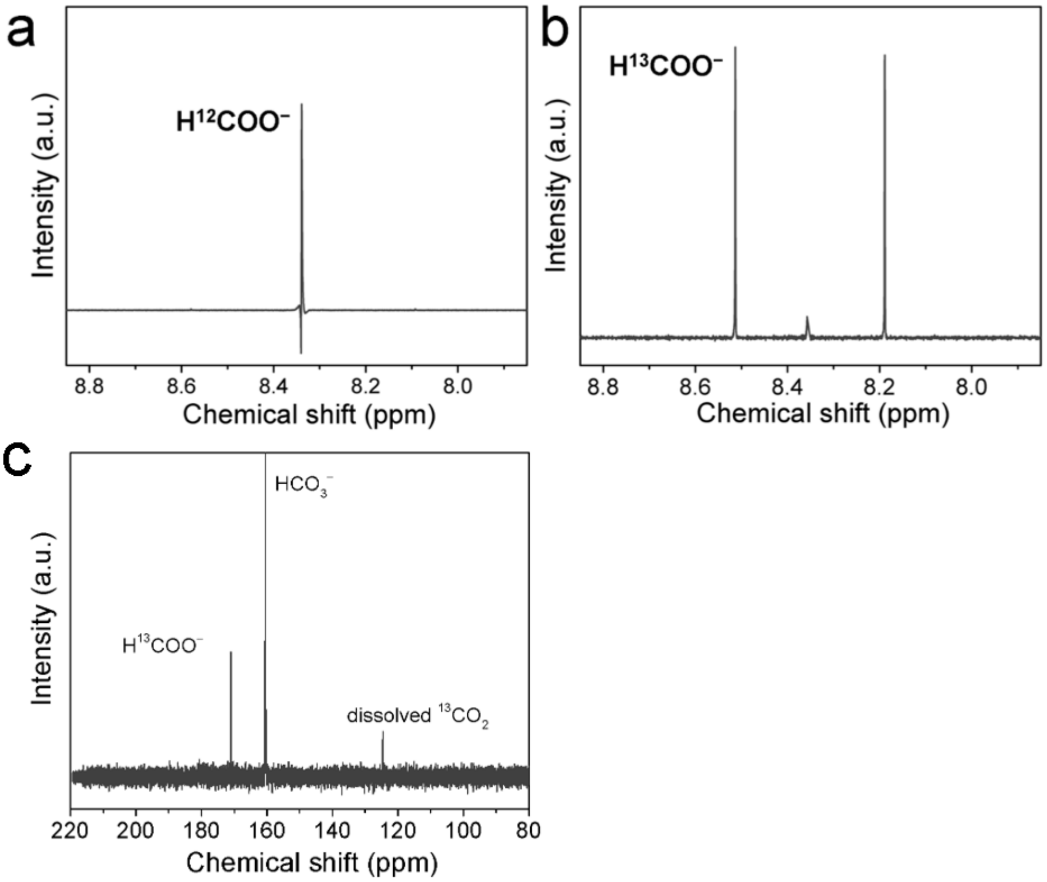

Supplementary Fig. 19 The NMR spectra of products. ^{1}H NMR spectra with different carbon sources. a) $^{12}CO_2$ and b) $^{13}CO_2$. c) The ^{13}C NMR spectra with $^{13}CO_2$ as carbon source during CO_2RR.

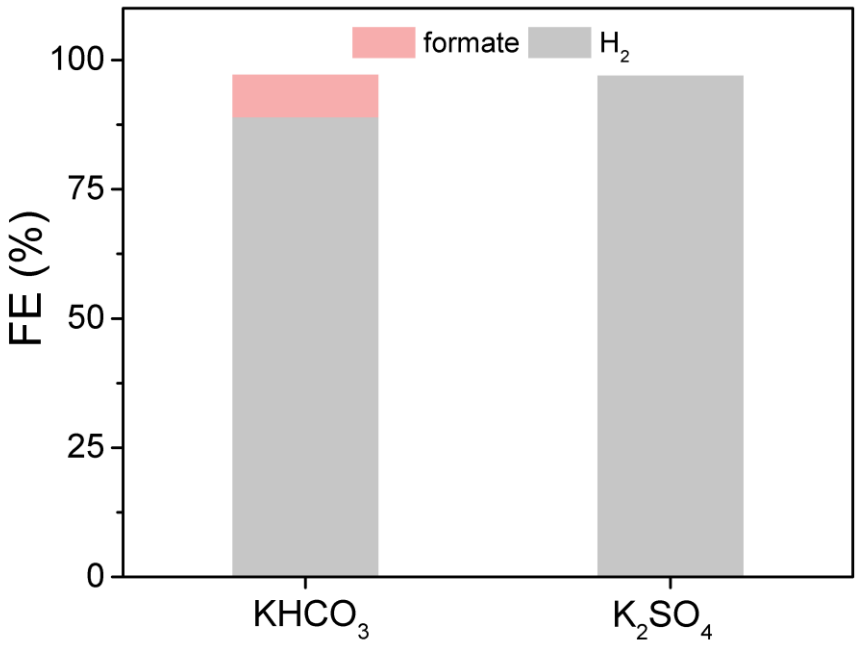

Supplementary Fig. 20 Electrochemical CO_2RR test in Ar-saturated 0.5 M $KHCO_3$ and Ar-saturated 0.5 M K_2SO_4 solution for 1 h. In Ar-saturated $KHCO_3$, small amount of formate can be detected which was from CO_2 decomposed by HCO_3^-. To exclude the effect of HCO_3^-, K_2SO_4 was selected and the production of formate is too low to be detected. This experiment proved that the carbon source is mainly from the additional CO_2 feed.

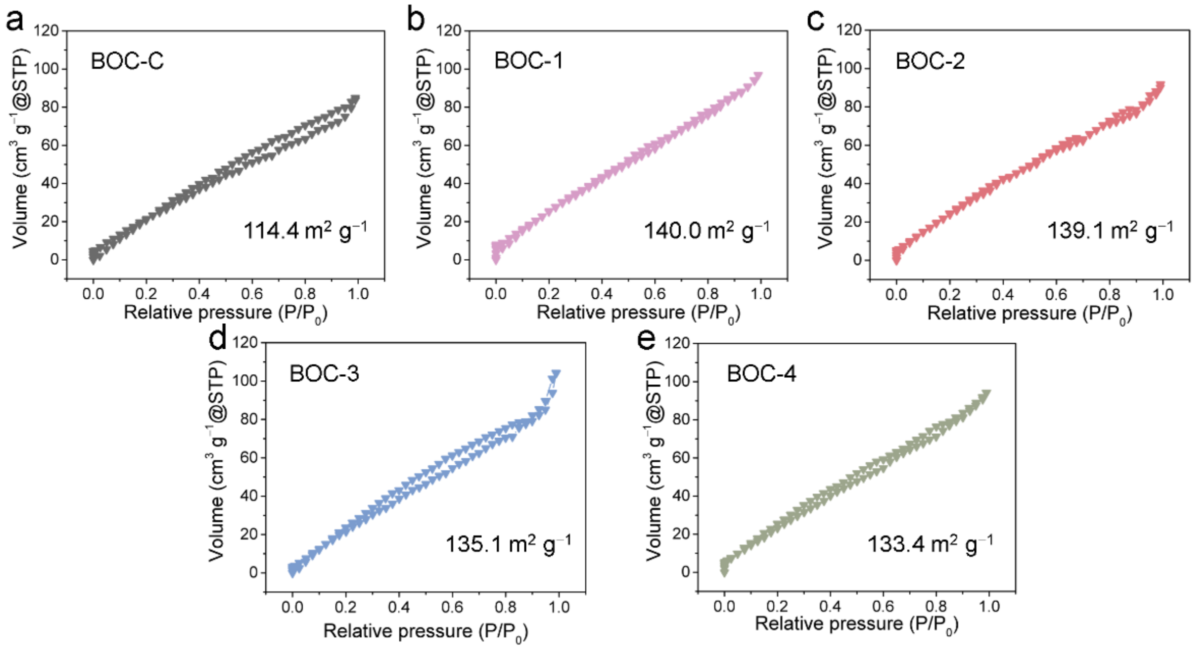

Supplementary Fig. 21 N_2 adsorption/desorption isotherms of a) BOC-C, b) BOC-1, c) BOC-2, d) BOC-3 and e) BOC-4. The corresponding BET surface are listed in the plot.

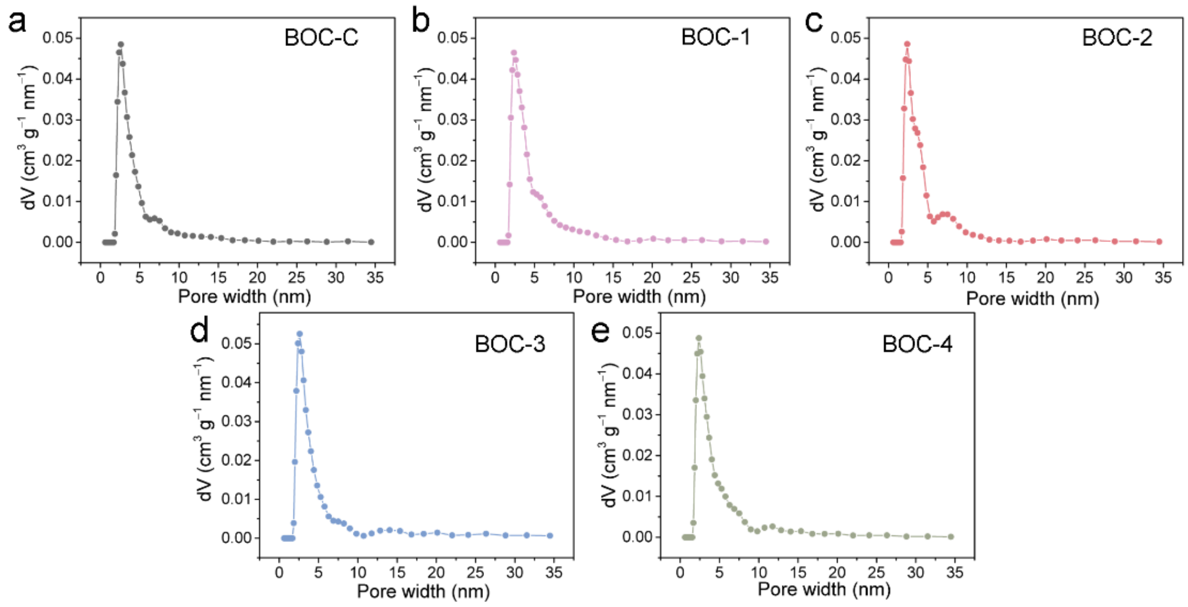

Supplementary Fig. 22 Pore size distributions of a) BOC-C, b) BOC-1, c) BOC-2, d) BOC-3 and e) BOC-4.

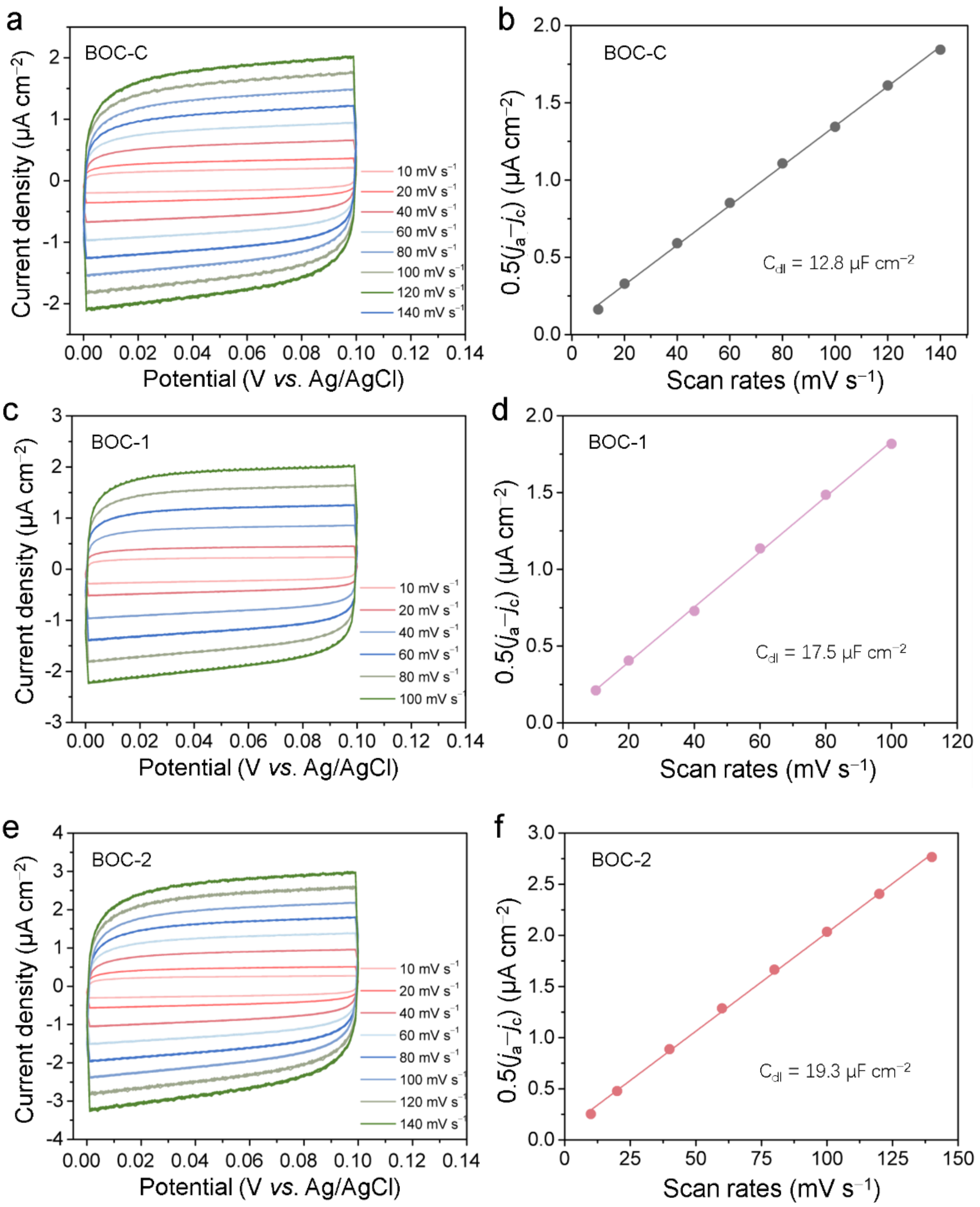

a
BOC-C
Current density (μA cm^{-2})
Potential (V vs. Ag/AgCl)
10 mV s^{-1}
20 mV s^{-1}
40 mV s^{-1}
60 mV s^{-1}
80 mV s^{-1}
100 mV s^{-1}
120 mV s^{-1}
140 mV s^{-1}
b
BOC-C
0.5(j_a−j_c) (μA cm^{-2})
Scan rates (mV s^{-1})
C_{dl} = 12.8 μF cm^{-2}
c
BOC-1
Current density (μA cm^{-2})
Potential (V vs. Ag/AgCl)
10 mV s^{-1}
20 mV s^{-1}
40 mV s^{-1}
60 mV s^{-1}
80 mV s^{-1}
100 mV s^{-1}
d
BOC-1
0.5(j_a−j_c) (μA cm^{-2})
Scan rates (mV s^{-1})
C_{dl} = 17.5 μF cm^{-2}
e
BOC-2
Current density (μA cm^{-2})
Potential (V vs. Ag/AgCl)
10 mV s^{-1}
20 mV s^{-1}
40 mV s^{-1}
60 mV s^{-1}
80 mV s^{-1}
100 mV s^{-1}
120 mV s^{-1}
140 mV s^{-1}
f
BOC-2
0.5(j_a−j_c) (μA cm^{-2})
Scan rates (mV s^{-1})
C_{dl} = 19.3 μF cm^{-2}

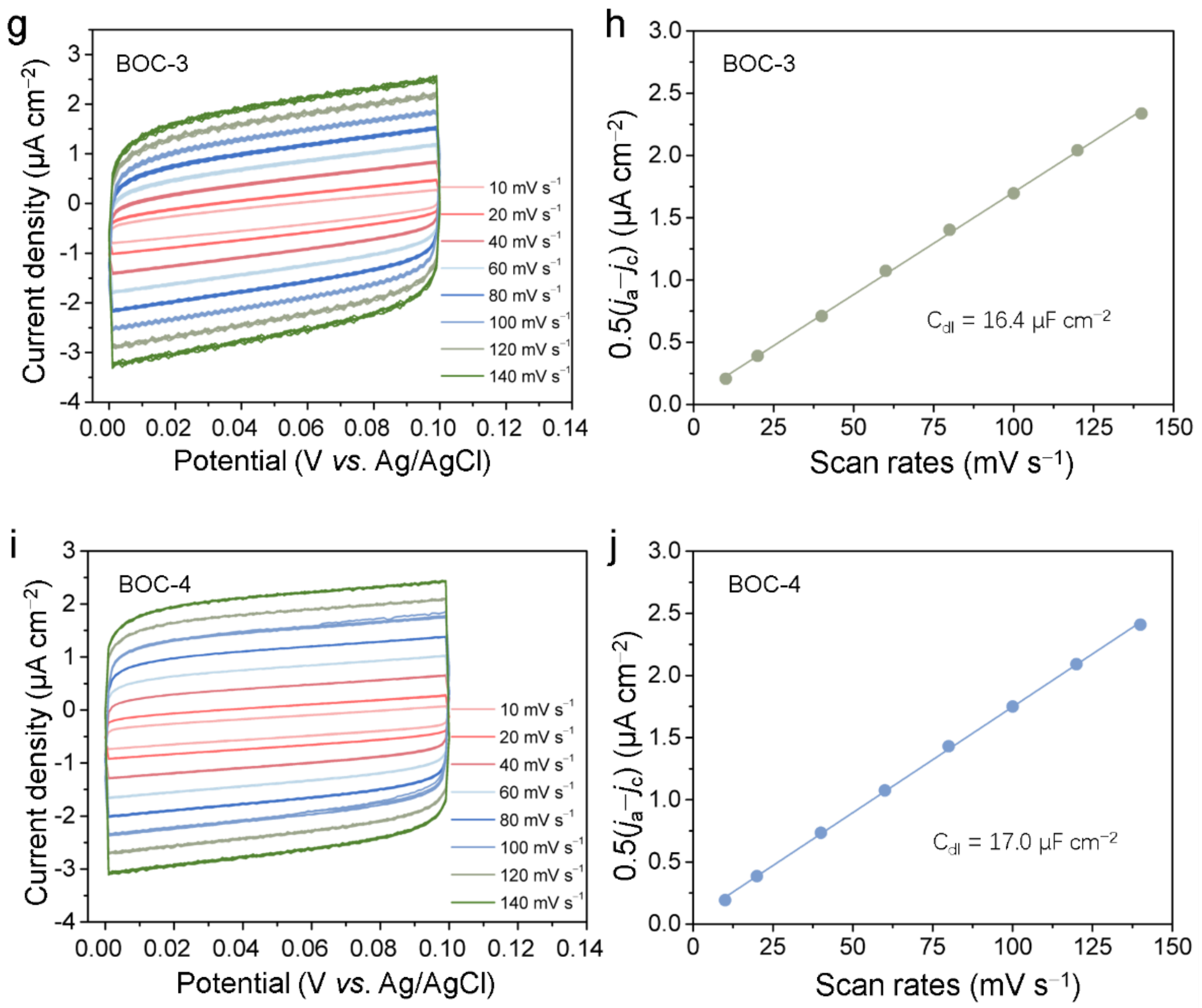

Supplementary Fig. 23 CV curves and the derived C_{dl} value of a), b) BOC-C, c), d) BOC-1, e), f) BOC-2, g), h) BOC-3 and i), j) BOC-4, respectively.

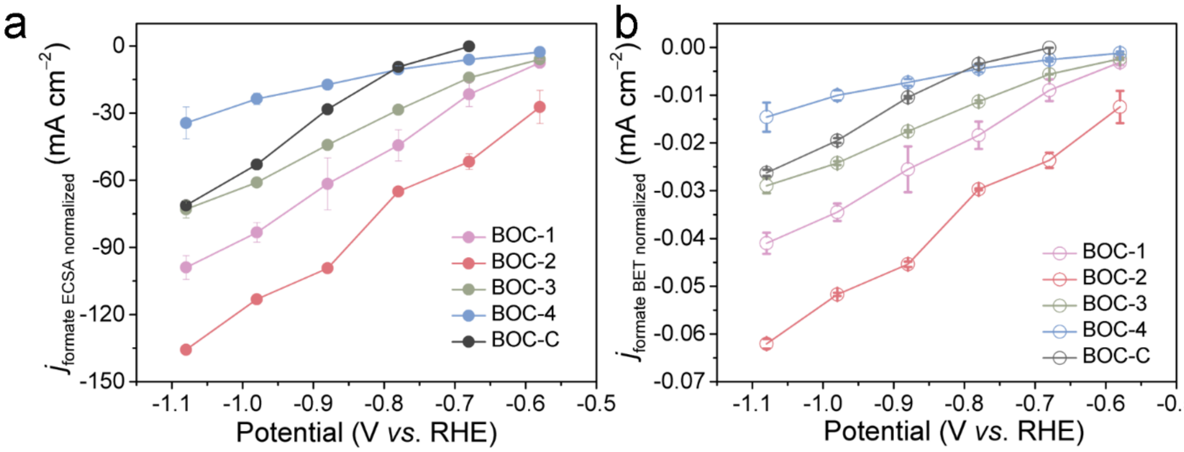

Supplementary Fig. 24 The partial current density of formate normalized by a) electrochemical surface area (ECSA) and b) specific surface area (BET) for different catalysts. The error bars represent the standard deviation of three independent experiments.

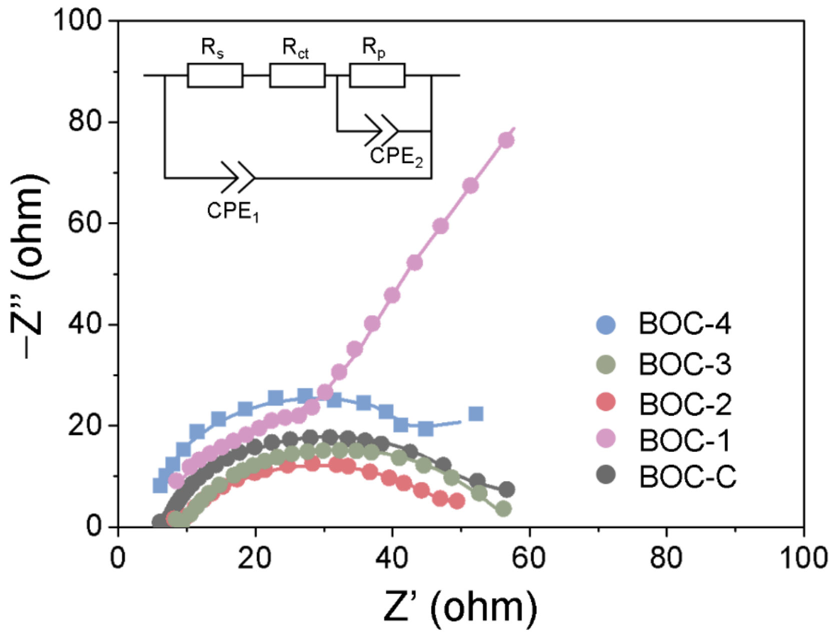

Supplementary Fig. 25 The EIS plot of different samples and the inset is the employed equivalent circuit. The circles represent the EIS data while the solid line represent corresponding fitted results. The model used here includes two sections: (1) the first is corresponding to the uncompensated solution resistance (R_s) while there are some variations in R_s for different samples owing to the roughness and experimental error such as electrode area, distance, temperature and so on; (2) the second section reflects interfacial resistance at electrode-electrolyte interface including charge transfer process (R_{ct}) and water and hydroxyl adsorption (R_p).

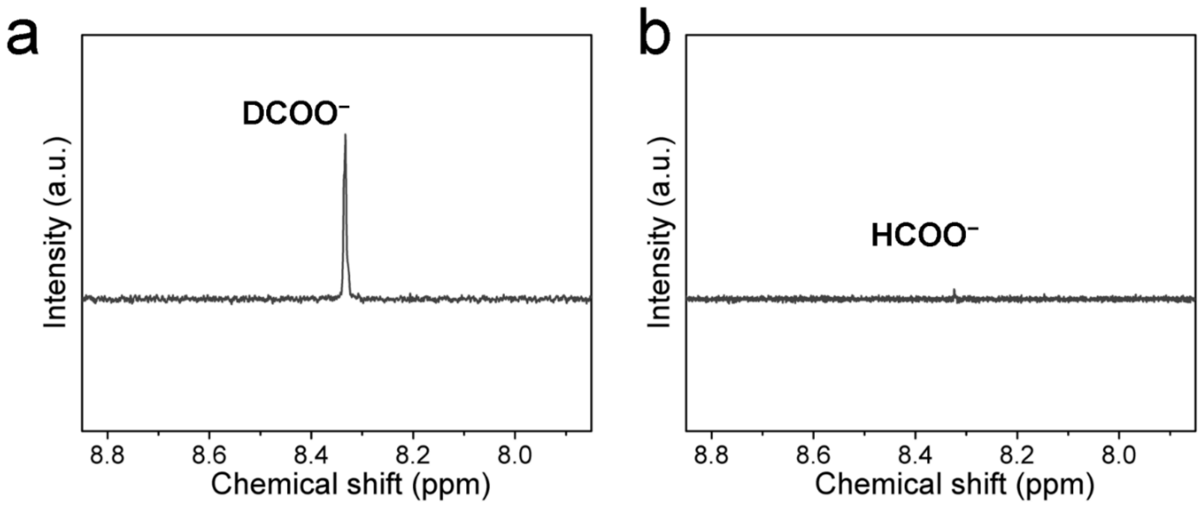

Supplementary Fig. 26 The investigation of the source of protons in CO_2RR to formate. a) ^{2}H NMR and b) ^{1}H NMR spectra for the liquid products collected from 0.5 M $KHCO_3$-D_2O electrolyte after electrolysis. The main signal of formate was from ^{2}H NMR while the signal of $HCOO^-$ in ^{1}H NMR was too low, suggesting that the proton source of formate is mainly from water rather than bicarbonate.

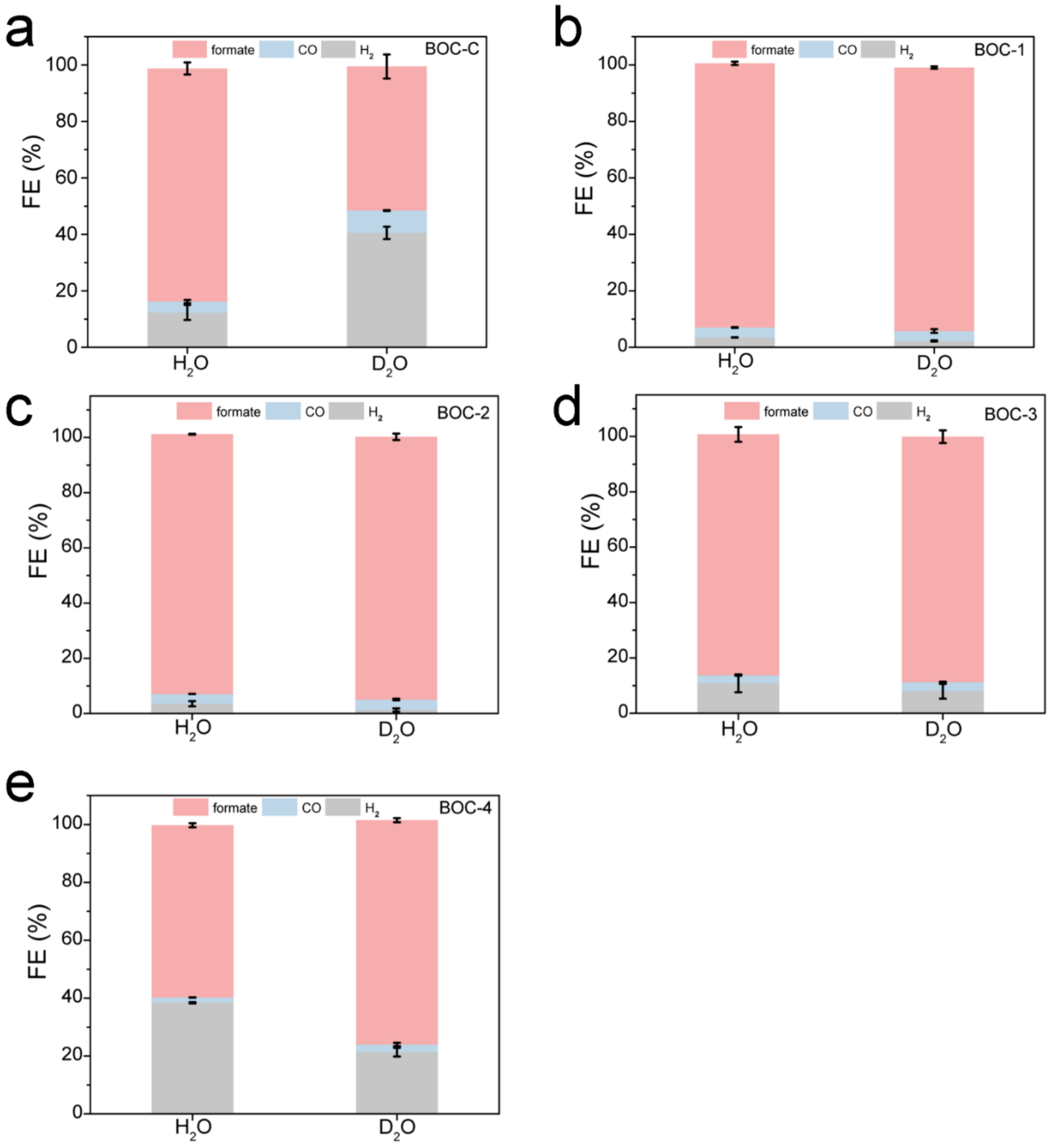

Supplementary Fig. 27 Effect of water dissociation for BOC samples. FE of formate, CO and H_2 in CO_2-saturated 0.5 M $KHCO_3$-H_2O and 0.5 M $KHCO_3$-D_2O electrolyte at −0.88 V for a) BOC-C, b) BOC-1, c) BOC-2, d) BOC-3 and e) BOC-4. The error bars represent the standard deviation of three independent experiments.

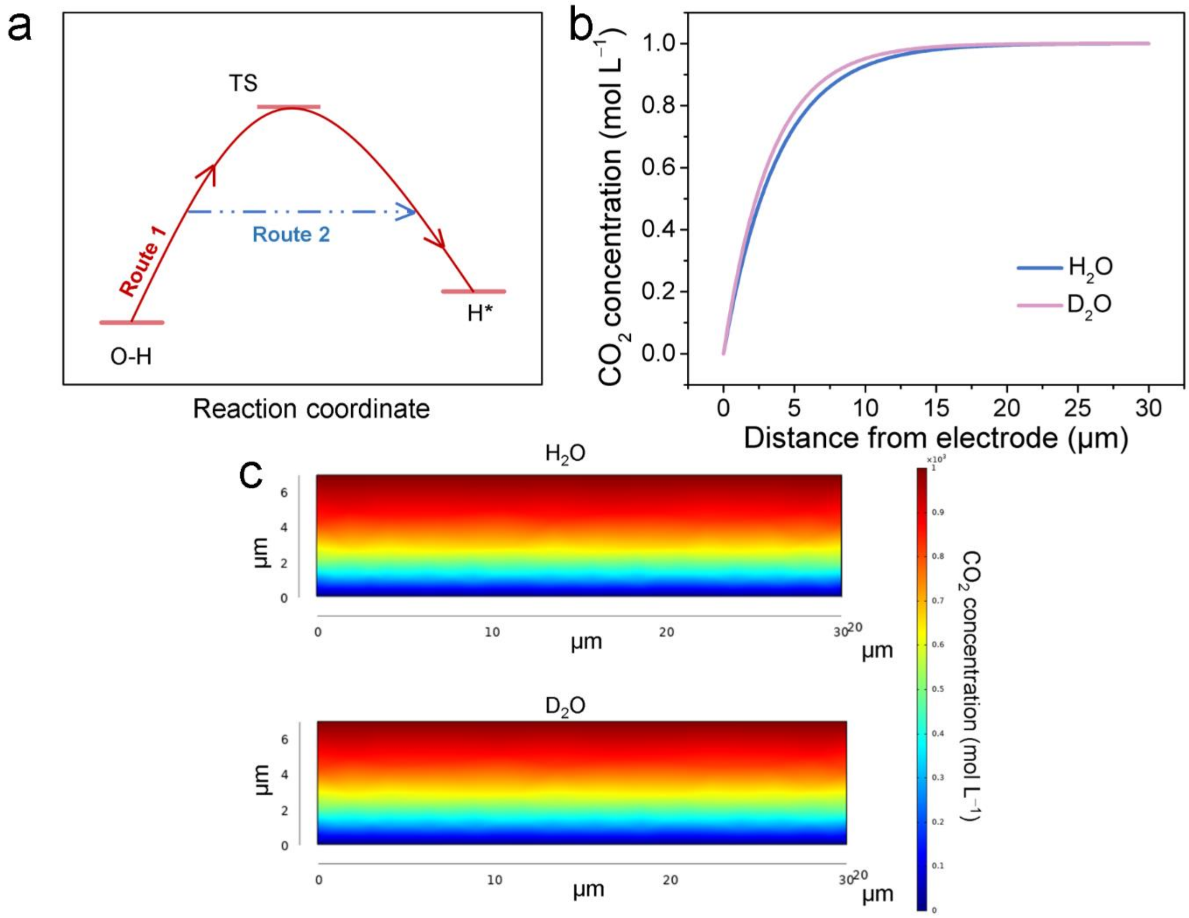

Supplementary Fig. 28 The effect of H_2O and D_2O in reaction rates. a) Schematic diagram of the H-OH cracking in water dissociation. b) CO_2 concentration against the distance from electrode in H_2O and D_2O. c) The finite element method based on simulation of CO_2 diffusion in H_2O (upper) and D_2O (bottom).

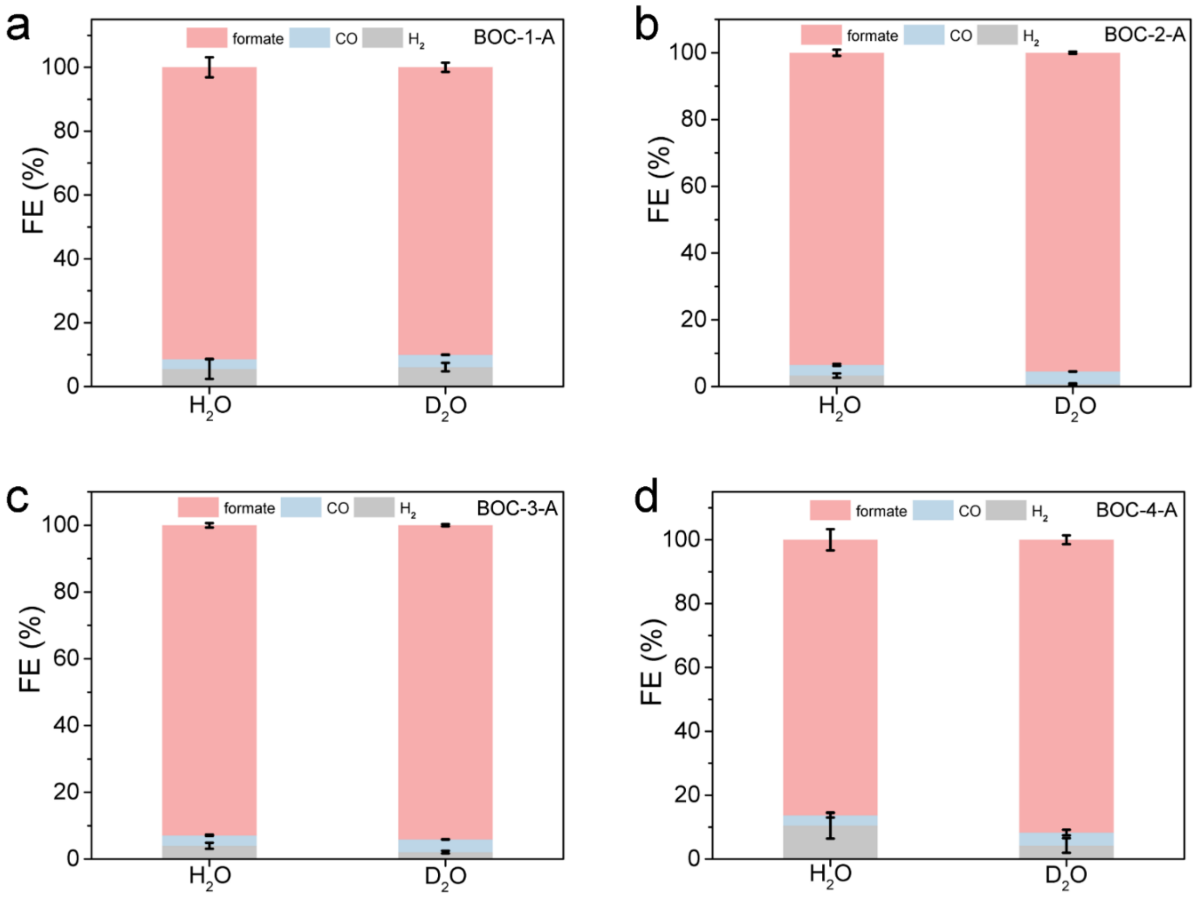

Supplementary Fig. 29 Effect of water dissociation in electrocatalysis for BOC samples after thermal treatment. FE of formate, CO and H_2 in CO_2-saturated 0.5 M $KHCO_3$-H_2O and 0.5 M $KHCO_3$-D_2O electrolyte at −0.88 V for a) BOC-1-A, b) BOC-2-A, c) BOC-3-A and d) BOC-4-A. The error bars represent the standard deviation of three independent experiments.

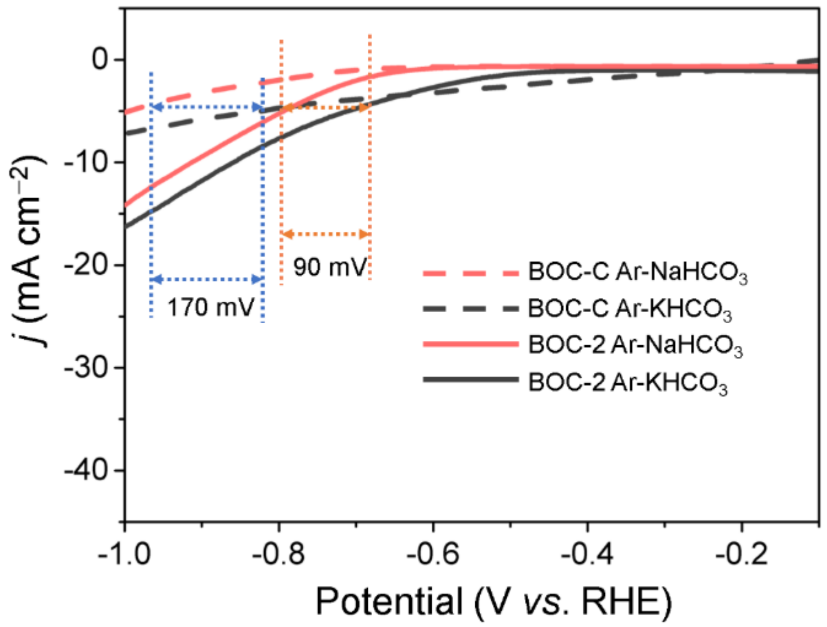

Supplementary Fig. 30 The LSV curves of BOC-C and BOC-2 in Ar-saturated 0.5 M $NaHCO_3$ and 0.5 M $KHCO_3$, respectively. K^+ is considered to be more effective in water dissociation relative to Na^+ and the overpotential of HER for BOC-C and BOC-2 are lower in $KHCO_3$ than that in $NaHCO_3$. However, less enhancement induced by cations can be observed on BOC-2, which can be attributed to the intrinsic faster water dissociation kinetics due to V_O introduction.

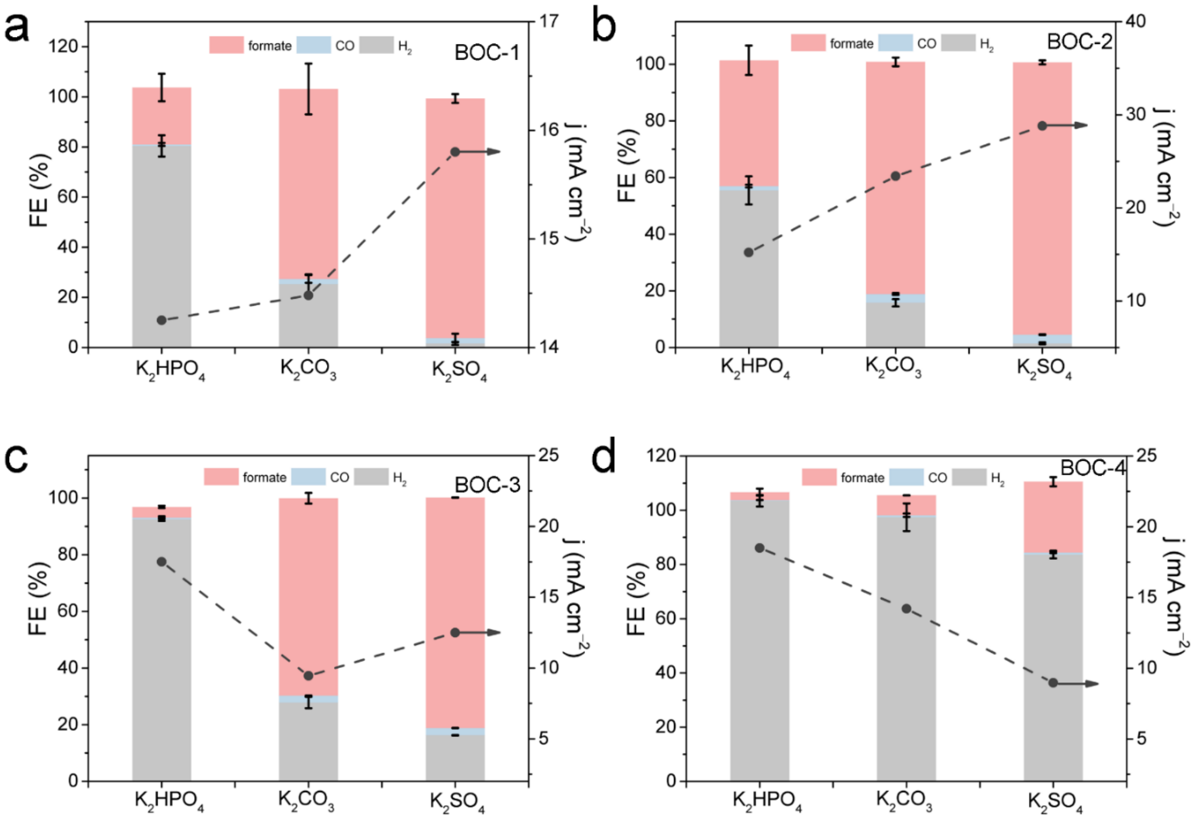

Supplementary Fig. 31 Effect of electrolyte with different local pH. FE of all products and current density at −0.78 V for a) BOC-1, b) BOC-2, c) BOC-3 and d) BOC-4. The three electrolyte with different buffer capacity and the same concentration of alkine metal cations were K_2HPO_4, K_2CO_3 and K_2SO_4, respectively. The local pH increased in the order of K_2HPO_4, K_2CO_3 and K_2SO_4. The error bars represent the standard deviation of three independent experiments.

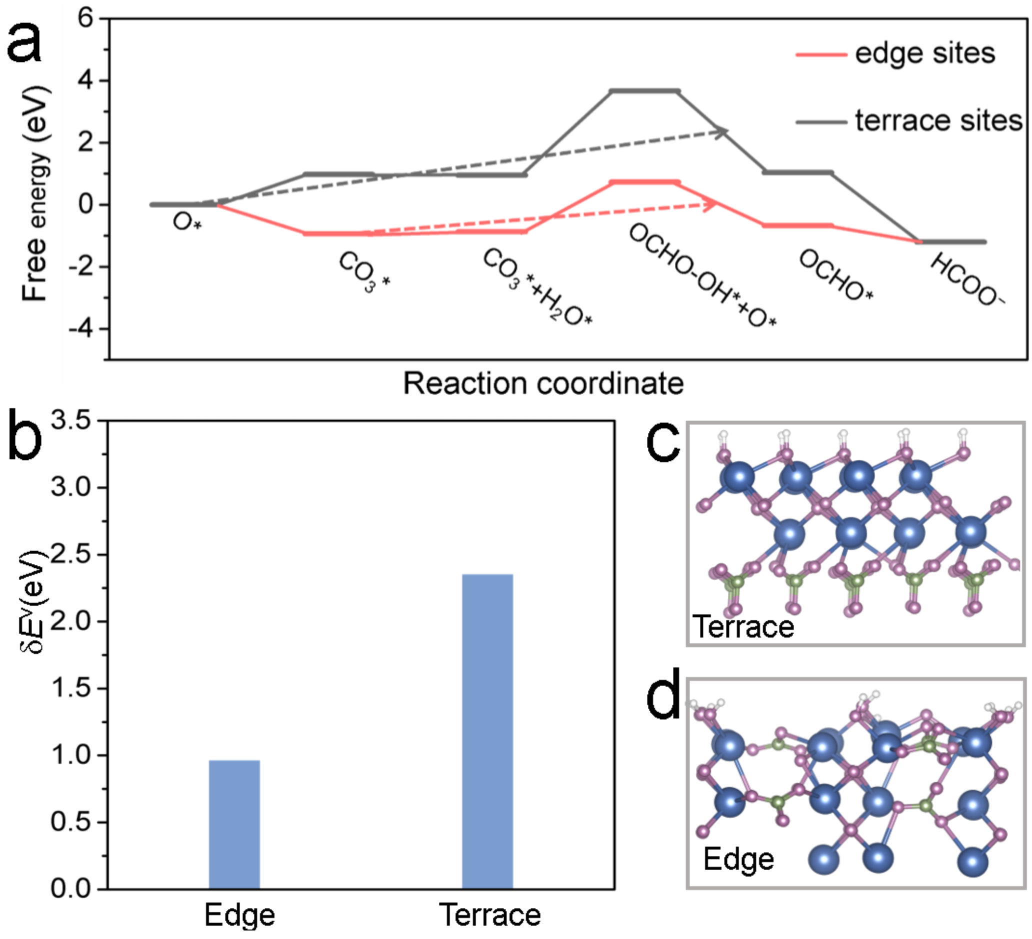

Supplementary Fig. 32 DFT calculations along the reaction pathway at edge sites and terrace sites. a) The FED of edge sites and terrace sites along the reaction route. The arrows plotted in the FED point from the *TDI* to the $TDTS^v$ (virtual transition states), and their difference ($TDI - TDTS^v$) are proved to be in direct proportion to the logarithm of TOF. b) The δE^v of BOC at edge sites and terrace sites. c), d) The corresponding model of BOC at terrace sites and edge sites.

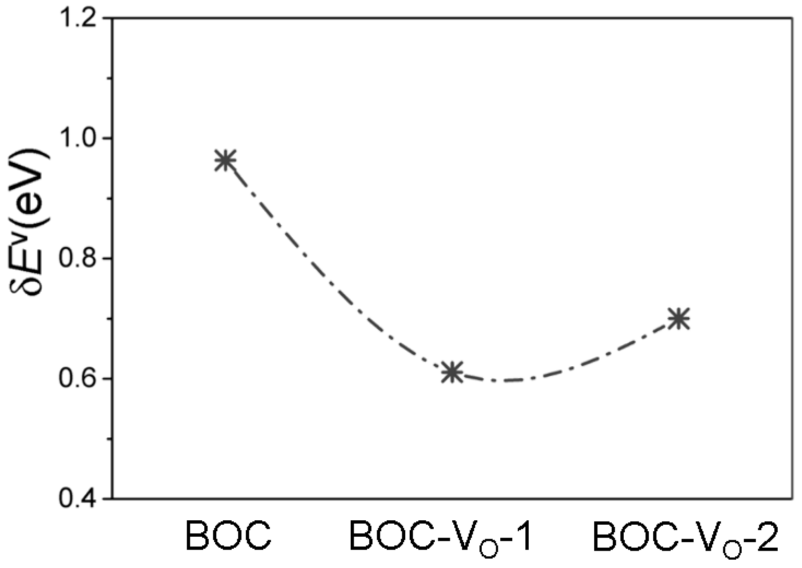

Supplementary Fig. 33 The δE^v of normal BOC, BOC-V_O-1 and BOC-V_O-2.

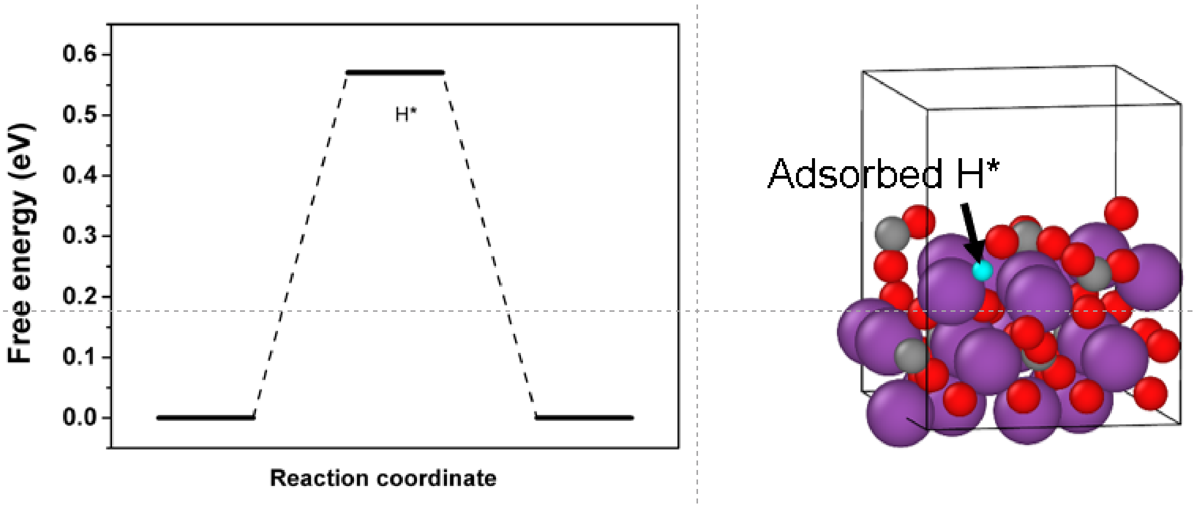

Supplementary Fig. 34 DFT calculations about H* adsorption on BOC with V_O (BOC-V_O-1 on the left) and the corresponding basic model (on the right). Colours in the model: purple balls are bismuth (Bi); red balls are oxygen (O); gray balls are carbon (C) and blue balls are added hydrogen (H*).

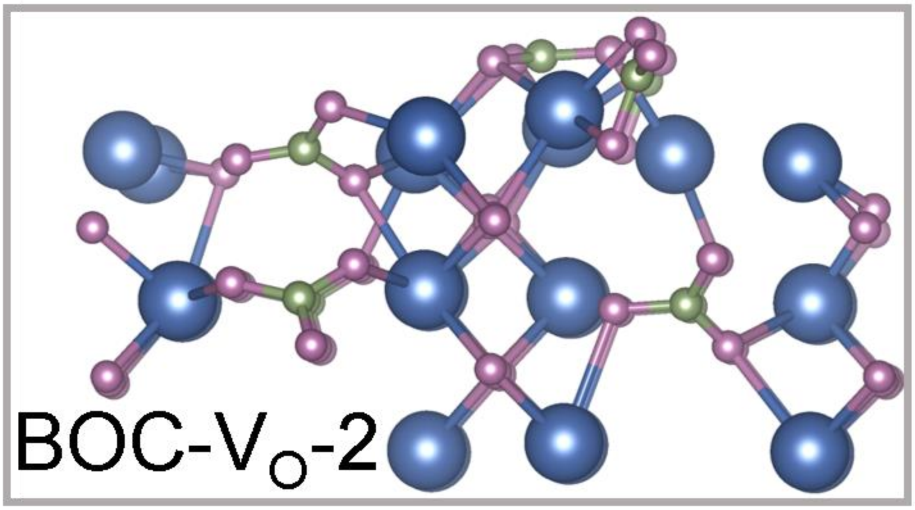

Supplementary Fig. 35 The basic model of BOC with more oxygen vacancy contents (BOC-V_O-2).

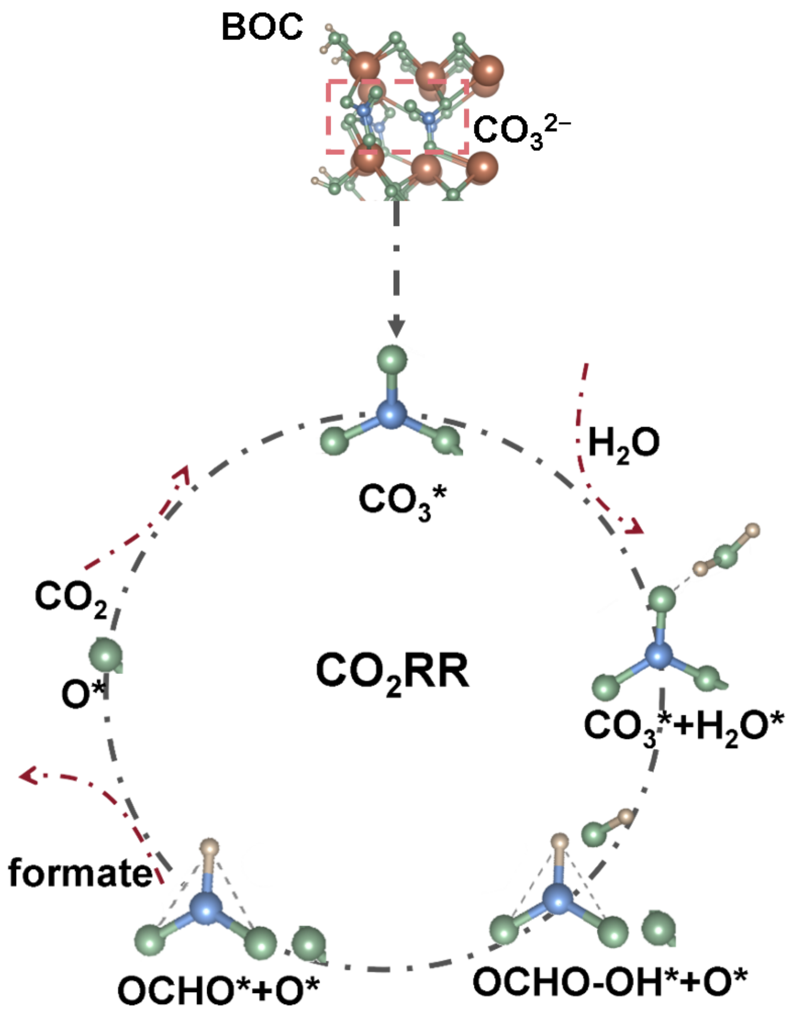

Supplementary Fig. 36 The schematic plot of catalytic cycle in CO_2RR based on DFT analysis. Among this, brown balls represent bismuth (Bi), green balls represent oxygen (O), blue balls represent carbon (C) and white balls represent hydrogen (H), respectively. During electroreduction, the intrinsic CO_3^{2-} in BOC interacted with electrolyte will spontaneously undergo charge rearrangement and be in-situ transformed into CO_3^* species. The CO_3* is more active and participates in CO_2RR to produce formate as the surface species. After that, one oxygen site was left and the input CO_2 can be easily adsorbed on oxygen site to form CO_3^* which proceeds the next cycle.

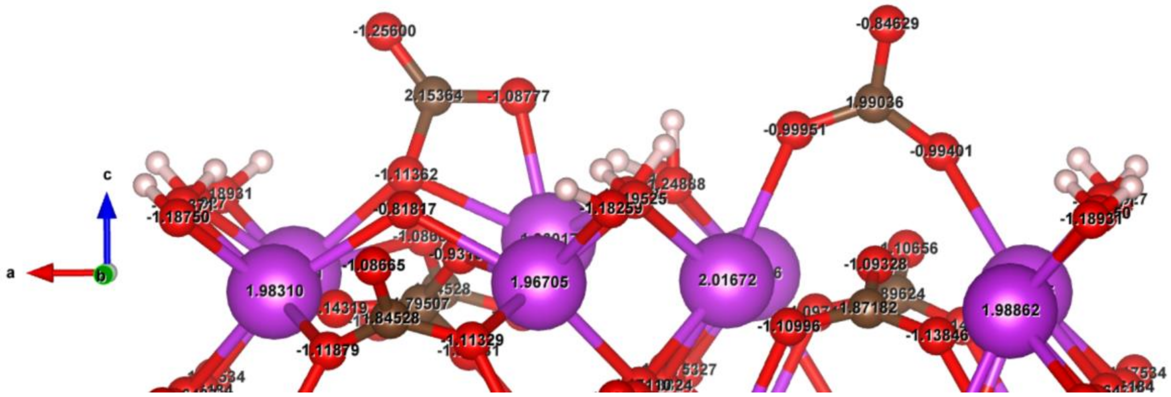

Supplementary Fig. 37 Bader charge distribution of surface species of CO_3*. The purple, red, brown, and white balls represent bismuth (Bi), oxygen (O), carbon (C) and hydrogen (H), respectively.

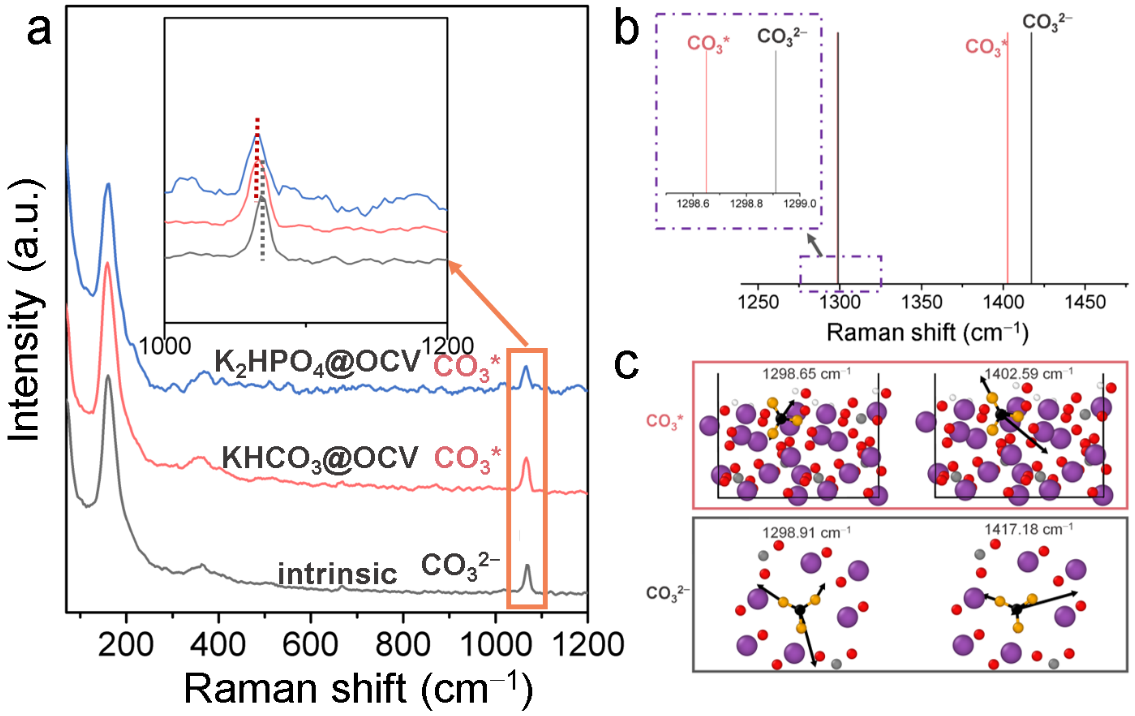

Supplementary Fig. 38 The identification of CO_3* and CO_3^{2-}. a) The Raman spectra of intrinsic BOC-2 and BOC-2 at OCV, where the wavenumber of CO_3* is slightly lower than that of CO_3^{2-}. b) Computed Raman vibrational features for CO_3^{2-} and CO_3*. c) The corresponding vibrational modes. The two major vibration modes were selected from more than 100 potential modes of vibration while the position of C-O vibration in CO_3* blueshifts mildly relative to that of CO_3^{2-}. The signal of C-O vibration of BOC at OCV is broaden and shifts towards low wavenumbers (1067 cm^{-1}) as compared with intrinsic signal of carbonate (1069 cm^{-1}) in BOC sample, which indicates the transformation of CO_3* from intrinsic CO_3^{2-}at least occurs under OCV. Apart from that, the signal at 1067 cm^{-1} is almost disappear accompanied with the appearance of $^{13}CO_3$* during $^{13}CO_2$ electroreduction (Supplementary Fig. 39). So, it is reasonable to conclude that the signal at 1067 cm^{-1} is at least mainly induced by CO_3* based on above analysis even though the contribution of intrinsic CO_3^{2-}cannot be totally ruled out.

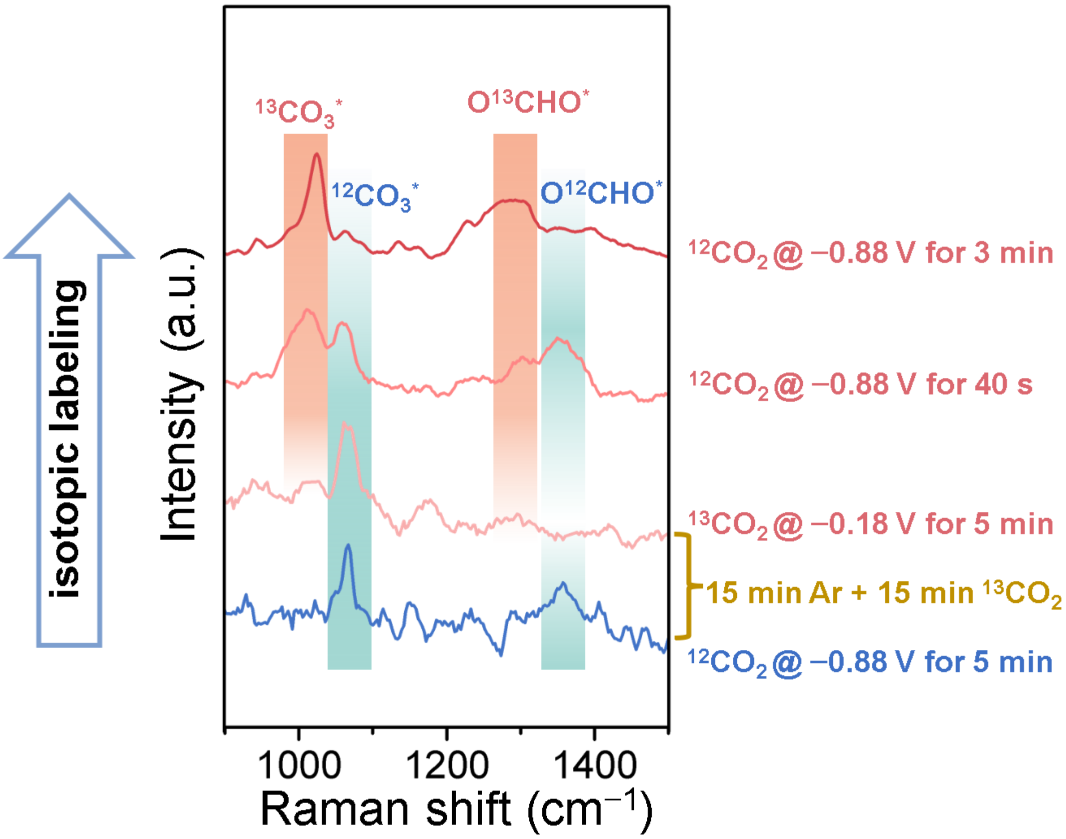

Supplementary Fig. 39 The in-situ Raman spectra of BOC with $^{12}CO_2$ and $^{13}CO_2$ as carbon source, respectively.

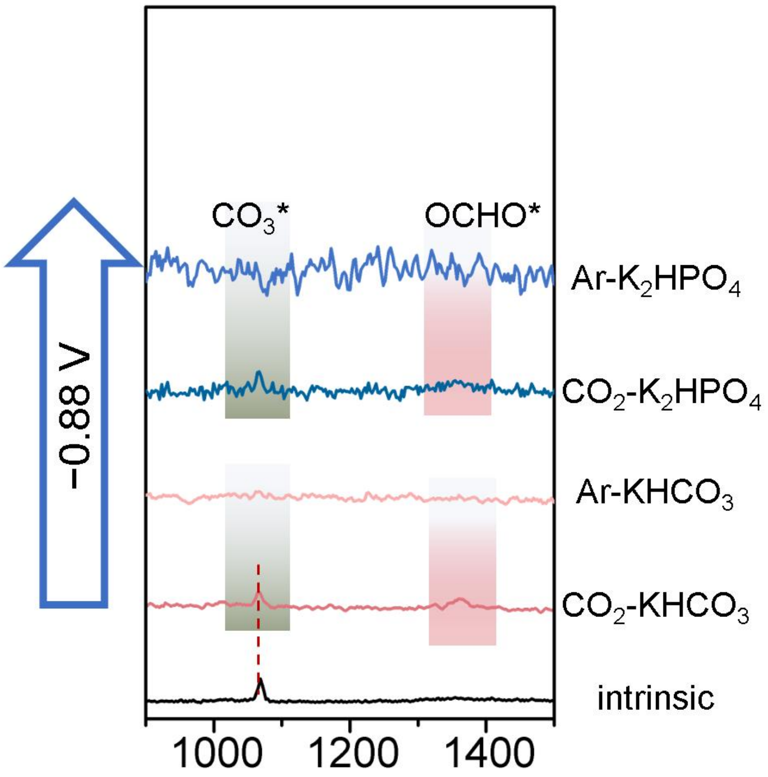

Supplementary Fig. 40 The in-situ Raman spectra of BOC-2 with CO_2-saturated 0.5 M $KHCO_3$, Ar-saturated 0.5 M $KHCO_3$, CO_2-saturated 0.5 M K_2HPO_4 and Ar-saturated 0.5 M K_2HPO_4 solutions as electrolyte, respectively. The green areas represent the characteristic signals of CO_3* and the red area is the signal of the OCHO* originated from CO_2RR. After replacing CO_2 by Ar, the intensity of CO_3* decreases notably with the disappear of OCHO* signal, indicating the involvement of CO_3* in formate production.

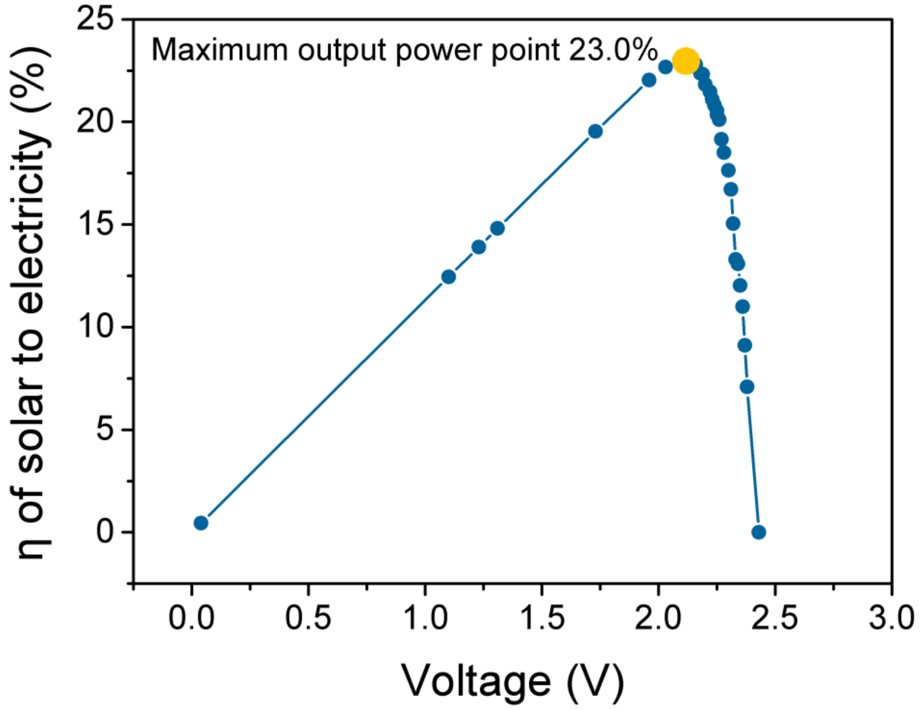

Supplementary Fig. 41 The solar to electricity conversion efficiency of the commercial three junction GaInP/GaInAs/Ge solar cell under AM 1.5 (100 mW cm^{-2}).

Supplementary Table 1 The atomic V_O concentration estimated by EPR.

Sample	BOC-1	BOC-2	BOC-3	BOC-4
V_O (at.%)	0.031	0.044	0.060	0.076

Supplementary Table 2 EXAFS fitting parameters at the Bi L_3 edge for BOC-1, BOC-2 and BOC-3. CN: coordination numbers; R: bond distance; σ^2: Debye-Waller factors; ΔE_0: the inner potential correction. R factor: goodness of fit.

Sample	Shell	CN	R(Å)	σ^2	ΔE_0	R factor
BOC-1	Bi-O	2.7	2.23	0.0024	-2.4	0.022
BOC-2	Bi-O	2.6	2.25	0.0026	-1.7	0.023
BOC-3	Bi-O	2.4	2.26	0.0037	-4.4	0.024

Supplementary Table 3 Electrochemical performance for CO_2RR to formate of Bi-based catalysts in H-cell reported recently.

Catalyst	Electrolyte	Potential (V vs. RHE)	$j_{formate}$ (mA cm^{-2})	$FE_{formate}$ (%)	Ref.
BOC NSs	0.1 M $KHCO_3$	−0.6	0.83	83	[1]
BOC NSs	0.5 M $NaHCO_3$	−0.7	9.35	85	[2]
P-BiOI	0.5 M $KHCO_3$	−1.5	81	96	[3]
Bi/CeOx	0.2 M Na_2SO_4	−1.3	149	90	[4]
MOF-derived BOC	0.5 M $KHCO_3$	−0.7	9.6	96	[5]
BOC NSs	0.5 M $KHCO_3$	−1.2	49.7	87	[6]
BOC NSs	0.5 M $KHCO_3$	−1.2	42.3	90	[7]
C-Bi RDs	0.5 M $KHCO_3$	−1.1	21.7	97	[8]
Bi NSs	0.1 M $KHCO_3$	−1.3	15	85	[9]
Bi-Sn	0.1 M $KHCO_3$	−1.0	9.3	94	[10]
Bi nanoribbons	0.5 M $KHCO_3$	−1.2	46.4	85	[11]
Bi_2O_3	0.5 M $KHCO_3$	−0.9	8	91	[12]

BOC with V_O	0.5 M $KHCO_3$	−0.9	32.5	94	This work

Supplementary Table 4 Electrochemical performance for CO_2RR to formate in flow-cell reported recently.

Catalyst	Electrolyte	Current density (mA cm^{-2})	TOF (s^{-1})	$FE_{formate}$ (%)	Ref.
Sn-N-C	0.5 M $KHCO_3$	200	1.4	50-60	[13]
BiO_n cluster	0.1 M KOH (MEA)	500	4.2	90	[14]
Bi_2O_3/BiO_2	0.5 M $KHCO_3$	114	0.27	98.12	[15]
BOC with V_O	1.0 M KOH	200	0.72	81	This work
SnO_2 NPs	1.0 M KOH	147	/	46	[16]
BOC with V_O	1.0 M KOH	1000	/	93	[6]
$Bi_{0.1}Sn$	1.0 M KOH	200	/	97	[17]
Bi_2O_3@C	1.0 M KOH	224	/	93	[18]
H-Sn_3O_4	1.0 M KOH	462	/	91	[19]
SnO_2/Sn	1.0 M KOH	114	/	94	[20]
Sn-In NPs	1.0 M KOH	236	/	94	[21]
SnS	1.0 M KOH	120	/	88	[22]
Bi-In alloy NPs	1.0 M KOH	250	/	97.8	[23]
MIL-68(In)-NH_2	1.0 M $KHCO_3$	114	/	94.4	[24]

$ZnIn_2S_4$	1.0 M $KHCO_3$	300	/	99.3	[25]

The turnover frequency (TOF) of BOC-2 was calculated according to the equation as follows:

$$\mathrm{TOF} = \frac{j \times FE_{formate}}{z\mathrm{F}n} \quad (1)$$

where j is the total current density; $\mathrm{FE_{formate}}$ is the corresponding Faradaic efficiency; z is the number of transfer electrons, which is 2; F is the Faraday constant (96485 C mol^{-1}); n is the mole of active sites and all Bi atoms in electrode were assumed to be active sites in our work. The n is calculated based on the equation as follows:

$$\mathrm{n} = \frac{x_{Bi} \times m_{cat.}}{\mathrm{M_{Bi}}} \quad (2)$$

where, $\mathrm{x_{Bi}}$ is the metallic composition in electrocatalyst, $\mathrm{m_{cat.}}$ is the mass of electrocatalyst and $\mathrm{M_{Bi}}$ is the relative atomic mass of Bi. The TOF cited in this Table is calculated based on this equation. For Ref. 13 and 14, the metallic composition is 8.8 wt% and 8.0 wt%, respectively, according to ICP-MS results. In our work, the metallic composition is considered as 82.0 wt% based on chemical formula.

Supplementary Table 5 The C_{dl} value and electrochemical surface area (ECSA) for BOC-C, BOC-1, BOC-2, BOC-3 and BOC-4.

	BOC-C	BOC-1	BOC-2	BOC-3	BOC-4
C_{dl} (μF cm^{-2})	12.8	17.5	19.3	16.4	17.0
ECSA (cm^2)	0.21	0.29	0.32	0.27	0.28

Supplementary Table 6 The equivalent circuit parameters in EIS analysis.

Sample	R_s (Ω)	R_{ct} (Ω)	R_p (Ω)
C-BOC	6.8	48.4	398.7
BOC-1	6.3	42.9	366.9
BOC-2	7.8	40.0	348.5
BOC-3	7.2	46.7	380.0
BOC-4	6.5	46.5	399.8

Supplementary Table 7 PV-EC CO_2RR to formate system reported recently.

Catalyst	Electrolyte	Full-cell potential (V)	j (mA cm^{-2})	$FE_{formate}$ (%)	STF (%)	Product	Ref.
mp Bi	0.5 M $NaHCO_3$	2.5	~3	/	1.5	formate^1	[26]
V_O rich N-SnO_2 NSs	0.1 M $NaHCO_3$	4.5	~3	55	/	formate	[27]
Ti/graphite/CS/MWCNTs/RuCP	0.4 M KPi	1.9	6.4	80	7.2	formate^1	[28]
Porous Bi	1 M $KHCO_3$	2.6	~10	95	8.5	formate^2	[29]
BiNN-CFs	1 M KOH	2.0	11.7	~80	13.3	formate^2	[30]
**BOC with V_O**	**0.5 M $KHCO_3$**	**2.1**	**8.8**	**93**	**13.3**	**formate^3**	**This work**
Co_2FeO_4	0.1 M $KHCO_3$	2.45	13.1	92.3	15.5	CO	[31]

CdS-CNT	0.1 M $KHCO_3$	5.03	/	~95	2.18	CO	[32]

The solar to fuel (STF) conversion efficiency reported in previous works is calculated according to the following equations:

For fomate[1]

$$\eta(\%) = \frac{P_{out}}{P_{in}} \times 100\% = \frac{\Delta rG_m^\theta \times n_{formate}}{P_{solar} \times S_{illuminated\ area}} \times 100\% \quad (3)$$

where ΔrG_m^θ = 270.138 kJ mol^{-1}, $n_{formate}$ represents the moles of produced formate, P_{solar} is the power of input solar (100 mW cm^{-2}) and $S_{illuminated\ area}$ is the irradiation area of PV.

$$\eta(\%) = \frac{P_{out}}{P_{in}} \times 100\% = \frac{J_{solar} \times FE_{product} \times S_{electrode} \times E^0_{product}}{P_{solar} \times S_{illuminated\ area}} \times 100\% \quad (4)$$

where J_{solar} means the current density in the working system, $FE_{product}$ is the corresponding Faradaic efficiency of specific product, $S_{electrode}$ is the geometric area of working electrode, $E^0_{product}$ is the thermodynamic cell potential for CO_2-H_2O to the specific product and P_{solar} is the power of input solar (100 mW cm^{-2}).

For formate[2], $E^0_{formate}$ is 1.43 V (1.23 V vs. RHE for OER and −0.20 V vs. RHE for CO_2RR to formate)

For formate[3], $E^0_{formate}$ is 1.25 V because the thermodynamic potential of CO_2RR to formate is pH-dependence and the $E^0_{formate}$ should be 1.25 V (1.23 V vs. RHE for OER and −0.02 vs. RHE V for CO_2RR to formate) in our system with CO_2-saturated 0.5 M $KHCO_3$ as electrolyte.

For CO, E^0_{CO} is 1.34 V (1.23 V for OER and −0.11 V vs. RHE for CO_2RR to CO)

Supplementary Table 8 The correction from the zero-point energy, entropy and heat capacity for converting the total energies to Gibbs free energies (units: eV)[33].

Species	TS (eV)	ZPE (eV)	$\int C_p dT$ (eV)
H_2(g)	0.27	0.42	0.09
H_2O(g)[a]	0.42	0.59	0.09
*O	0	0.08	0
*CO	0.08	0.22	0.05
*CO_2	0	0.58	0
*OCHO	0.2	0.63	0.1
*CO_3	0	1.12	0
*OH	0	0.15	0

[a]We used gas-phase H_2O at 0.035 bar as the reference state because at this pressure liquid water is equilibrium with gas-phase H_2O at 300 K.

References

1. Lv, W. et al. $Bi_2O_2CO_3$ nanosheets as electrocatalysts for selective reduction of CO_2 to formate at low overpotential. *ACS Omega* **2**, 2561-2567 (2017).
2. Zhang, Y. et al. Controllable synthesis of few-layer bismuth subcarbonate by electrochemical exfoliation for enhanced CO_2 reduction performance. *Angew. Chem. Int. Ed.* **57**, 13283-13287 (2018).
3. Liu, P. F., Zu, M. Y., Zheng, L. R. & Yang, H. G., Bismuth oxyiodide microflower-derived catalysts for efficient CO_2 electroreduction in a wide negative potential region. *Chem. Commun.* **55**, 12392-12395 (2019).
4. Duan, Y. X. et al. Boosting production of HCOOH from CO_2 electroreduction via Bi/CeO_x. *Angew. Chem. Int. Ed.* **60**, 8798-8802 (2021).
5. Yuan, W. W. et al. In situ transformation of bismuth metal–organic frameworks for efficient selective electroreduction of CO_2 to formate. *J. Mater. Chem. A* **8**, 24486-24492 (2020).
6. Fan, T. et al. Achieving high current density for electrocatalytic reduction of CO_2 to formate on bismuth-based catalysts. *Cell Rep. Phys. Sci.* **2**,100353 (2021).
7. Wang, Y. et al. Sub-2 nm ultra-thin $Bi_2O_2CO_3$ nanosheets with abundant Bi-O structures toward formic acid electrosynthesis over a wide potential window. *Nano Res.* **15**, 2919-2927 (2021).
8. Xie, H. et al. Facet engineering to regulate surface states of topological crystalline insulator bismuth rhombic dodecahedrons for highly energy efficient electrochemical CO_2 reduction. *Adv. Mater.* **33**, e2008373 (2021).
9. Yao, D. et al. The controllable reconstruction of Bi-MOFs for electrochemical CO_2 reduction through electrolyte and potential mediation. *Angew. Chem. Int. Ed.* **60**, 18178-18184 (2021).

10. Wu, Z. et al. Engineering bismuth-tin interface in bimetallic aerogel with a 3D porous structure for highly selective electrocatalytic CO_2 reduction to HCOOH. *Angew. Chem. Int. Ed.* **60**, 12554-12559 (2021).
11. Li, Y. et al. In situ confined growth of bismuth nanoribbons with active and robust edge sites for boosted CO_2 electroreduction. *ACS Energy Lett.* **7**, 1454-1461 (2022).
12. Deng, P. et al. Bismuth oxides with enhanced bismuth–oxygen structure for efficient electrochemical reduction of carbon dioxide to Formate. *ACS Catal.* **10**, 743-750 (2019).
13. Duarte, M. et al. Enhanced CO_2 electroreduction with metal-nitrogen-doped carbons in a continuous flow reactor. *J. CO_2 Util.* **50**, 101583 (2021).
14. Jiang, X. et al. Boosting CO_2 electroreduction to formate via bismuth oxide clusters. *Nano Res.* 1-8 (2022) DOI: 10.1007/s12274-022-5073-0.
15. Feng, X. et al. Bi_2O_3/BiO_2 nanoheterojunction for highly efficient electrocatalytic CO_2 Reduction to formate. *Nano Lett.* **22**, 1656-1664 (2022).
16. Liang, C. et al. High efficiency electrochemical reduction of CO_2 beyond the two-electron transfer pathway on grain boundary rich ultra-small SnO_2 nanoparticles. *J. Mater. Chem. A*. **6**, 10313 (2018).
17. Li, L. et al. Stable, active CO_2 reduction to formate via redox modulated stabilization of active sites. *Nat. Commun*. **12**, 5223 (2021).
18. Deng, P. et al. Metal–organic framework-derived carbon nanorods encapsulating bismuth oxides for rapid and selective CO_2 electroreduction to formate. *Angew. Chem. Int. Ed.* **59**, 10807–10813 (2020).
19. Liu, L. et al. Tuning Sn_3O_4 for CO_2 reduction to formate with ultra-high current density. *Nano Energy* **77**, 105296 (2020).

20. Ning, S. et al. Electrochemical reduction of SnO_2 to Sn from the bottom: in-situ formation of SnO_2/Sn heterostructure for highly efficient electrochemical reduction of carbon dioxide to formate. *J. Catal.* **399**, 67–74 (2021).

21. Wang, J. et al. In-Sn alloy core-shell nanoparticles: In-doped SnO_x shell enables high stability and activity towards selective formate production from electrochemical reduction of CO_2. *Appl. Catal. B: Environ.* **288**, 119979 (2021).

22. Zou, J., Lee, C. & Wallace, G. Boosting formate production from CO_2 at high current densities over a wide electrochemical potential window on a SnS catalyst. *Adv. Sci.* **8**, 2004521 (2021).

23. Yao, K. et al. Metal-organic framework derived dual-metal sites for electroreduction of carbon dioxide to HCOOH. *Appl. Catal. B: Environ.* **311**, 121377 (2022).

24. Wang, Z. et al. Efficient electroconversion of carbon dioxide to formate by a reconstructed amino-functionalized indium–organic framework electrocatalyst. *Angew. Chem. Int. Ed.* **60**, 19107–19112 (2021).

25. Chi, L. et al. Stabilizing indium sulfide for CO_2 electroreduction to formate at high rate by zinc incorporation. *Nat. Commun*. **12**, 5835 (2021).

26. Yang, H. et al. Selective CO_2 reduction on 2D mesoporous Bi nanosheets. *Adv. Energy Mater.* **8**, 201801536 (2018).

27. Li, Z. et al. Elucidation of the synergistic effect of dopants and vacancies on promoted selectivity for CO_2 electroreduction to formate. *Adv. Mater.* **33**, e2005113 (2021).

28. Kato, N. et al. A large-sized cell for solar-driven CO_2 conversion with a solar-to-formate conversion efficiency of 7.2%. *Joule* **5**, 687-705 (2021).

29. Piao, G., Yoon, S. H., Han, D. S. & Park, H. Ion-enhanced conversion of CO_2 into formate on porous dendritic bismuth electrodes with high efficiency and durability. *ChemSusChem* **13**, 698-706 (2020).

30. Wulan, B. et al. Electrochemically driven interfacial transformation for high-performing solar-to-fuel electrocatalytic conversion. *Adv. Energy Mater.* **12**, 202103960 (2022).

31. Mi, Y. et al. Cobalt–iron oxide nanosheets for high-efficiency solar-driven CO_2–H_2O coupling electrocatalytic reactions. *Adv. Funct. Mater.* **30**, 202003438 (2020).

32. Qin, B. et al. Efficient electrochemical reduction of CO_2 into CO promoted by sulfur vacancies. *Nano Energy* **60**, 43-51 (2019).

33. Nørskov, J. K. et al. Origin of the overpotential for oxygen reduction at a fuel-cell cathode. *J. Phys. Chem. B* **108**, 17886-17892 (2004).
